# Synthesis, characterization and theoretical studies of nitroxoline azo dye metal complexes and their role in mitigation of rheumatoid arthritis

**DOI:** 10.1038/s41598-025-06518-4

**Published:** 2025-06-20

**Authors:** Hoda A. El-Ghamry, Mohamed Gaber, Mariam S. Napolion, Amira Atta, Tarek M. Mohamed, Nadia A. El‐Wakiel

**Affiliations:** 1 Chemistry Department, Faculty of Science, Tanta University, Tanta, 31527 Egypt; 2 Biochemistry Division, Chemistry Department, Faculty of Science, Tanta University, Tanta, 31527 Egypt

**Keywords:** Nitroxoline, 2,6-Dichloroaniline, Azo dye, Metal complexes, Adenosine deaminase, Rheumatoid arthritis, Biochemistry, Chemical biology, Drug discovery, Chemistry

## Abstract

In this work, an azo dye ligand of 2,6-dichloroaniline with nitroxoline (**CPAQ**), and its Zn(II), Cu(II), Cd(II), Ni(II) and Co(II) complexes have been synthesized. The structures of the synthesized compounds have been elucidated applying analytical and spectral tools. According to these results, all complexes proved to have a tetrahedral structure, except Ni(II) complex, which has an octahedral geometry. Analytical results also inferred the formation of Co(II) and Ni(II) complexes in the molar ratio 1M:1L and the remaining complexes in 1M:2L molar ratio. For further insight into the complexes’ geometry, bond lengths, bond angles, and electronic characteristics with respect to the organic ligand were assigned in addition to DFT calculations. HOMO and LOMO calculations show the Co(II) complex is more reactive. The interactions of the target compounds with the *Mus musculus* ADA enzyme structure (PDB ID: 1a4m) were estimated by applying molecular docking studies. The inhibitory effect of the synthesized compounds on adenosine deaminase enzyme (ADA) activity was tested in-vitro, showing the Co(II) to be the most active.

**Supplementary Information:**

The online version contains supplementary material available at 10.1038/s41598-025-06518-4.

## Introduction

In addition to being utilized in other significant applications, synthetic and natural azo compounds are an essential source of drug prototypes and a starting point for drug discovery. Additionally, and because of their ease of synthesis, high molar extinction coefficient, and wet fastness properties, azo dyes are an important class of organic compounds^1,2^. Mono-azo dyes are the most significant type of azo dyes^3^. They have many applications in different fields such as memory and recording devices^4,5^, molecular switches^6,7^, thermochromic^8^, catalysis^9,10^, supramolecular systems^11,12^, halographic data storage materials^13^, and metal sensors^14^, and textile and fiber dyeing, which mainly due to their capacity for adsorption and the effectiveness of their depletion brought about by the presence of the N=N group^15^. The remarkable biological activity of azo ligands with O and N donor atoms is well-known, with their remarkable chelating ability to all kinds of metal ions^1^. So, complex compounds containing several metal ions can be created using azo ligands. However, the properties of the produced complexes rely on the type of metal ion used^16^. Azo ligand complexes have a variety of uses in medicine, including antitumor^9^, antimicrobial^9,17^ and anti-inflammatory agents^18^. The study of azo ligand complexes is still in progress. These intricate molecules have many possible applications, and further study is required to completely comprehend their characteristics and potential applications^9^. Furthermore, pyridone, pyrazolone, pyrimidine, thiophene, quinoline, and indole derivatives are heterocyclic coupling components that are crucial for sophisticated industrial applications^19^. Among the important heterocyclic couplers for azo compounds is quinoline and its most important derivative 8-Hydroxyquinoline is due to its ability to chelate significant metal ions^20^. 8-Hydroxyquinoline and its derivatives have high antibacterial activities^21^. Furthermore, azo compounds obtained from 8-hydroxyquinoline derivatives are essential chelating agents for a variety of metal ions^22–27^. Because of their biological significance, ability to coordinate, and application as metal-extracting agents, quinoline and its derivatives’ chemical characteristics have been extensively discussed^28^. Their therapeutic qualities have drawn particular attention to them. Numerous quinoline derivatives are antimalarial and antiallergic medicines with chemotherapeutic action^29^. They may be used to treat a range of infections and exhibit broad-spectrum effectiveness against several herpes viruses^30^. Also, as an important derivative of 8-hydroxyquinoline, is nitroxoline (NIT) or 5-nitro-8-hydroxyquinoline, which is considered an antibiotic that is not classified under any known antibacterial class^31,32^. It is an effective urinary antibacterial agent against sensitive gram-positive and gram-negative organisms frequently encountered in urinary tract infections. NIT has fungistatic properties as well^33^. Additionally, angiogenesis and the growth of human bladder cancer are treated with it^34^.

According to recent research, the metal complexes of nitroxoline azo dye can be used as catalysts to remove color and degrade certain organic textile dyes^35^, corrosion inhibition of silicate glass^36^, dyeing polyester fabrics^37^ and function as antimicrobial agents^38^.

The chronic autoimmune inflammatory disease known as rheumatoid arthritis (RA) is characterized by joint destruction, systemic consequences, and synovial membrane inflammation. Its causes are multifaceted, involving genetic, environmental, and immunological elements. Among the various biochemical pathways linked to RA, the purinergic signalling pathway, especially the role of adenosine and its metabolites has gained significant interest. Adenosine deaminase (ADA) is an enzyme that facilitates the conversion of adenosine to inosine, playing a crucial role in regulating extracellular adenosine levels. Understanding the relationship between ADA and RA is essential for grasping the disease’s pathophysiology and for developing potential treatments^39^.

Adenosine is a nucleoside that tends to accumulate in the extracellular space during pathological conditions, such as tissue injury and inflammation. Since adenosine can influence the activation and proliferation of multiple immune cell types, such as T lymphocytes, dendritic cells, and macrophages, its immunomodulatory effects are significant. When adenosine binds to its receptors (A1, A2A, A2B, and A3), it can elicit anti-inflammatory responses, lowering the synthesis of cytokines that promote inflammation and encouraging the development of regulatory T cells^40^.

In the context of rheumatoid arthritis, adenosine levels are frequently altered, contributing to the inflammatory environment in affected joints. Although increased adenosine in inflamed tissues may help counter excessive inflammation, dysregulation of adenosine metabolism can worsen immune dysfunction, resulting in chronic inflammation and joint damage^41^.

ADA has a crucial function in maintaining adenosine homeostasis. By converting adenosine to inosine, ADA helps regulate the levels of these metabolites in the extracellular environment. Research has shown that ADA activity is significantly altered in RA. Higher ADA levels have been detected in the synovial fluid of RA patients, correlating with disease activity and joint inflammation^42^. This suggests that elevated ADA activity may lead to lower adenosine levels, which could reduce its anti-inflammatory effects and aggravate the autoimmune response characteristic of RA^43^.

Additionally, ADA has been linked to the regulation of T cell responses in RA. Increased ADA activity may promote T cell proliferation and cytokine production, further fuelling the inflammatory processes seen in RA^44^. Conversely, inhibiting ADA activity has been shown to elevate adenosine levels, which can foster anti-inflammatory responses and potentially mitigate joint inflammation and damage in experimental RA models^45^. The complex relationship between ADA and RA provides opportunities for new therapeutic approaches. Targeting ADA activity to adjust adenosine levels may be a promising strategy for managing RA. For instance, ADA inhibitors or agents that enhance adenosine signalling could provide dual benefits: reducing inflammation while facilitating the resolution of immune responses. Ongoing research is examining various ADA inhibitors and their potential clinical applications, with preliminary findings indicating positive effects on reducing disease activity in RA patients^46^. There are numerous previous reports on ADA inhibitors showing considerable activity such as natural plant extracts^43,47^ or metal ions^48^. As a result of the previous survey, we decided in this research to synthesize new Zn(II), Cu(II), Cd(II), Ni(II) and Co(II) complexes based on azo dye of 2,6-dichloroaniline with nitroxoline. The structure of synthesized complexes were elucidated by different analytical techniques. The interactions of the target compounds (the ligand and complexes) with the *Mus musculus* ADA enzyme structure (PDB ID: 1a4m), which causes rheumatoid arthritis, were estimated by applying the molecular docking studies. The inhibitory effect of the synthesized compounds on adenosine deaminase enzyme (ADA) activity was tested in-vitro.

## Experimental

### Materials, methods and equipments

Sigma-Aldrich supplied all analytical-grade reagents required for this work, which were utilized without additional purification. The supporting documentation included comprehensive details regarding the tools and methods utilized for structural affirmation are represented in detail in the supplementary file.

### Synthesis of azo dye ligand (CPAQ)

**Fig. 1:**
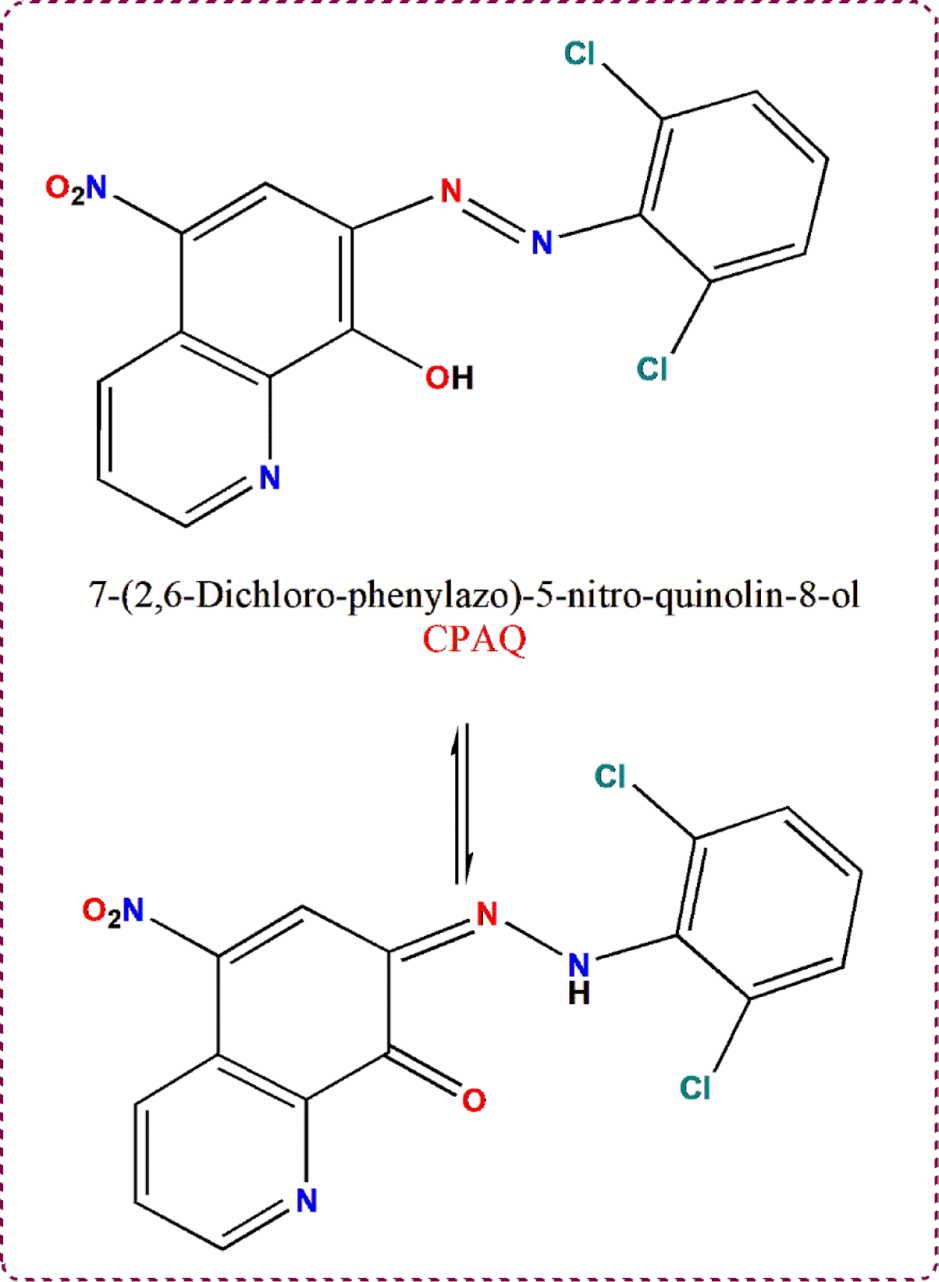
Tautomeric structures of the ligand **CPAQ.**

The targeting ligand, 7-((2,6-Dichloro-phenylazo)-5-nitro-quinoline-8-ol (symbolled as **CPAQ**, Fig. 1), was made by dissolving 1.77 g (10.73 mmol) of 2,6 Dichloro aniline in 25 ml of distilled water with 2.7 mL (12 M) of concentrated hydrochloric acid. The resultant solution was then cooled to 0 °C while being stirred. To create the diazonium chloride, 0.74 g of dissolved sodium nitrite (10.75 mmol) was carefully added to the prior solution in an ice-cold solution. The mixture was then stirred for 30 min in an ice bath. Finally, dropwise addition of the resultant solution to a solution that contained 2.04 g of 8-hydroxyl 5-nitro quinolone (10.73 mmol) dissolved in 10 mL of pyridine was performed. The orange dye that was generated was kept for two hours in an ice bath while being stirred. After filtering off the precipitate, it was rinsed thoroughly with distilled water and allowed to completely dry. The purity of the ligand was confirmed by TLC.

### Synthesis of the metal complexes

The ligand described above (i.e. **CPAQ**) was utilized in the synthesis of the Zn(II), Cu(II), Cd(II), Ni(II) and Co(II) complexes by dissolving 1.5 mmol of each ZnNO_3_.6H_2_O (0.446 g), CuCl_2_.2H_2_O (0.255 g), CdCl_2_ (0.274 g), NiCl_2_.6H_2_O(0.356 g), CoCl_2_.6H_2_O (0.356 g) in 15 mL of hot methanol. Each of these solutions was dropwise added to a hot methanolic solution containing 1.5 mmol of **CPAQ** (0.544 g). 5 drops of triethylamine were added to the formed mixtures which showed a sudden color change. The reaction mixtures were kept under reflux with stirring for 4 h to complete the precipitation of colored products which are probably the desired metal complexes. The formed precipitates were filtered off, washed thoroughly with hot methanol followed by ether, and kept for drying in a vacuum desiccator. The synthesized metal complexes were symbolled as **CPAQ-Zn, CPAQ-Cu, CPAQ-Cd, CPAQ-Ni and CPAQ-Co**.

### Quantum chemical computations

Additional information regarding the ligand’s and complexes’ shape, bond length, bond angle, and electronic properties can be obtained through the theoretical calculations performed by using the DMOL^3^ module adopted in the Materials Studio package. Within DMOL^3^ module, GGA- PBE functional,^49^ and employing the double numerical basis set with polarization functions (DNP)^50^.

### In‑silico study

Mus musculus ADA enzyme structure in three dimensions was taken from the Protein Data Bank (PDB ID: 1a4m). MOE-Dock 2014 software was used to perform a molecular docking study^51^. The chemical structures of the studied chemicals were sketched using the MOE builder, and they were minimized using the program force field MMFF94x. Subsequently, hydrogen atoms were added, undesirable water molecules were removed, and the protein was created. The 3D conformers were then docked using rescoring 1 (London dG) and rescoring 2 (GBVI/WSA dG). Using the "Lig- and Interactions" tool, which showed the different interactions that were established, the 2D protein–ligand interactions were visualized.

### Determination of ADA enzyme activity and protein

#### Materials and methods

##### Animals and ethical approval

Two adult Wistar Albino male rats (150–170 g; 6 weeks) were obtained from the Faculty of Veterinary Medicine, Alexandria University, Egypt, and housed for a week in a 20–22 °C controlled environment with a 12-h light–dark cycle for adaptation. Before the rats’ sacrifice, they were anesthetized with 0.3 mL/100 m of 10% chloral hydrate intraperitoneally. Euthanasia was carried out in compliance with institutional ethical rules by administering an overdose of the same anesthetic drugs. All experimental procedures involving animals were conducted under institutional and international guidelines for the care and use of laboratory animals. The Institutional Animal Care and Use Committee (IACUC) of the Faculty of Science, Tanta University reviewed and approved the study protocol under approval number IACUC-SCI-TU-0161 which is in accordance with ARRIVE guidelines. All efforts were made to minimize animal suffering and to use one of the animals required to achieve scientific validity.

##### Tissues

Joint tissue samples were collected immediately from adult Wistar male rats (150–170 g) were obtained from the Faculty of Agriculture, Alexandria University (Alexandria, Egypt)The tissues were cut off, rinsed with normal saline, and kept frozen at (− 20 °C) until analyzed^43^. Our study was carried out according to the guidelines approved by the Research Ethical Committee (Faculty of Science, Tanta University, Egypt) (IACUC-SCI-TU-0161).

##### Tissue homogenate preparation

Using a Teflon pestle homogenizer, 1 g of rat joints were homogenized in 10 ml of 50 mM sodium phosphate buffer, pH 7.5, containing 150 mM sodium chloride, to create the crude extract. After centrifuging the homogenate for 20 min at 13,200 g to exclude insoluble debris, it was ultrasonically sonicated for 20 min, 5 min off, and 5 min on. The supernatant was then classified as a crude extract ^43^.

#### Enzyme activity in rat joints

By combining 200 mg of joint tissue with 1.5 ml of 50 mM potassium phosphate buffer (pH 7.5) that contained 150 mM sodium chloride, the crude ADA enzyme was extracted. At 4 °C, a Teflon pestle homogenizer was used for this. The homogenate underwent a 15-min centrifugation at 8000 × g and a 15-min sonication at 4 °C. After that, the crude ADA enzyme-containing supernatant was gathered, and its enzymatic activity was assessed. ADA activity was assessed using a modified version of the method^43^. The reaction mixture contained 10% of the joint homogenate, 50 mM potassium phosphate buffer (pH 7.5), and 21 mM adenosine substrate solution. After 15 min of incubation at 37 °C, the enzyme reaction was stopped by adding a phenol/sodium nitroprusside solution (106 mM phenol; 0.17 mM sodium nitroprusside) and an alkaline hypochlorite solution (11 mM NaOCl; 125 mM NaOH). This was followed by another 15 min of incubation at 37 °C. The measurement of absorbance took place at 628 nm. The Bradford technique was used to calculate the protein content ^52^.

#### Purification and kinetic inhibition of ADA enzyme in rat joint

The enzyme was purified according to El-said^43^ in Biochemistry lab Faculty of Science following the reported method^48,53^. The complexes under investigation were introduced to the partly purified ADA enzyme at different doses (0.1–1 mM), incubated for one hour, and then their activity was assessed. Metal complex concentrations that inhibited ADA by 40%, 50%, and 60% were chosen, added to adenosine at different concentrations ((5, 10, 20, 35, 50, 75, 100 and 150 µM/L), and the enzyme activity was assessed. The measurements of Ki (Inhibition constant), Vmax, and Km were determined in accordance with Mohamed^48^.

## Results and discussion

### Structure affirmation

#### Molar conductivity and stoichiometry of metal complexes

According to the presumed molecular formulas, the azo ligand (**CPAQ**) and its five complexes, **CPAQ-Zn, CPAQ-Cu, CPAQ-Cd, CPAQ-Ni**, and **CPAQ-Co**, have good matching elemental analysis results (Table 1). According to these results, the molar ratios for the complexes **CPAQ-Zn, CPAQ-Cu**, and **CPAQ-Cd** were 1:2 (M:L), while the two complexes **CPAQ-Ni** and **CPAQ-Co** were 1:1 (M:L). The produced compounds are all readily and fully soluble in highly polar solvents like DMSO and DMF, while they are hardly or not soluble in non-polar and low polar solvents. The findings demonstrating the non-electrolytic nature of complexes came from the molar conductance measurements made for a 10^–3^ M solution in DMF solvent, which showed values fell between 10.3 and 15.2 Ω^−1^ cm^2^^54,55^.

**Table 1:** The molecular weights, microanalysis, colours, molecular formulae, empirical formulae, and molar conductance data of **CPAQ** ligand and its metal chelates.

No	Molecular formula (Empirical formulae)	M.p. (^°^C) (M. Wt.)	Colour (Λ_m_)	Microanalysis found (Calc.) %			
				% C	% H	% N	% M*
CPAQ	(C_15_H_8_Cl_2_N_4_O_3_) L	250 (363.16)	Orange (–)	49.87 (49.61)	2.47 (2.22)	15.60 (15.43)	–
CPAQ-Zn	[C_30_H17Cl4N8O7.5Zn) [Zn(L)_2_]·1.5H_2_O	270 (816.71)	Orange (11.8)	44.30 (44.12)	2.31 (2.1)	13.91 (13.72)	8.83 (8.01)
CPAQ-Cu	[C_30_H15Cl4CuN8O6.5] [Cu(L)_2_]·0.5H_2_O	276 (796.85)	Brown (13.5)	44.95 (45.22)	2.17 (1.90)	14.08 (14.06)	8.30 (7.97)
CPAQ-Cd	[C_30_H14CdCl4N8O6] [Cd(L)_2_]	Over 300 (836.71)	Orange (12.5)	43.27 (43.06)	1.85 (1.69)	13.62 (13.39)	12.84 (13.43)
CPAQ-Ni	[NiLCl(H_2_O)_3_] C_15_H13Cl3N4NiO6	270 (510.34)	Orange (15.2)	35.49 (35.30)	2.65 (2.57)	11.15 (10.98)	11.52 (11.50)
CPAQ-Co	[C_15_H9Cl3CoN4O4] [CoLCl(H_2_O)]	243 (474.55)	Reddish orange (10.3)	38.2 (37.96)	2.08 (1.91)	11.93 (11.81)	12.06 (12.42)

*Calculated from TG thermograms.

#### El-mass spectra

**Fig. 2:**
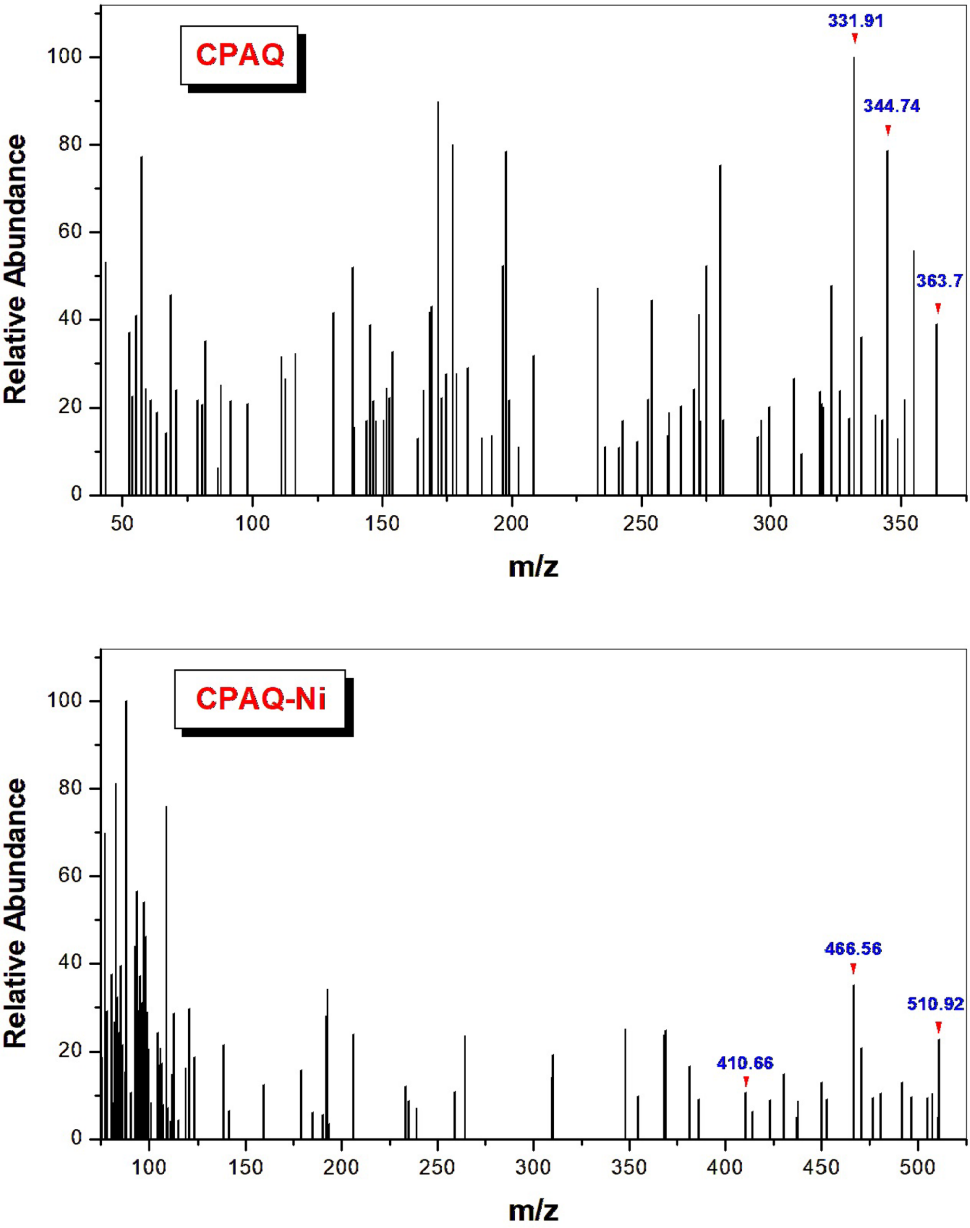
Mass spectra of **CPAQ** ligand and **CPAQ-Ni** complex.

The results of the mass spectral analysis utilizing the EI mass spectral technique have been used to determine the molecular weight of the ligand under investigation and its metal complexes. The ligand’s and metal complexes’ mass spectra are shown in Figs. 2 & supplementary file. As shown, the molecular ion peak observed in the ligand spectrum at m/z 363.72 is consistent with the proposed molecular weight (calculated: 363.16). Fragmentation of the **CPAQ** structure supported the formation of the ligand in the proposed structure. Investigation of the complexes’ spectra showed the molecular ion peaks at 816.84, 796.75, 836.07, 510.92 and 474.20 for **CPAQ-Zn**, **CPAQ-Cu**, **CPAQ-Cd**, **CPAQ-Ni** and **CPAQ-Co**, respectively, which are in good agreement with concluded molecular weights that were calculated to be 816.71, 796.85, 836.71, 510.34 and 474.55, respectively. The proposed fragmentation pathways of the metal complexes are represented in the supplementary file.

#### IR spectra and mode of bonding

**Fig. 3:**
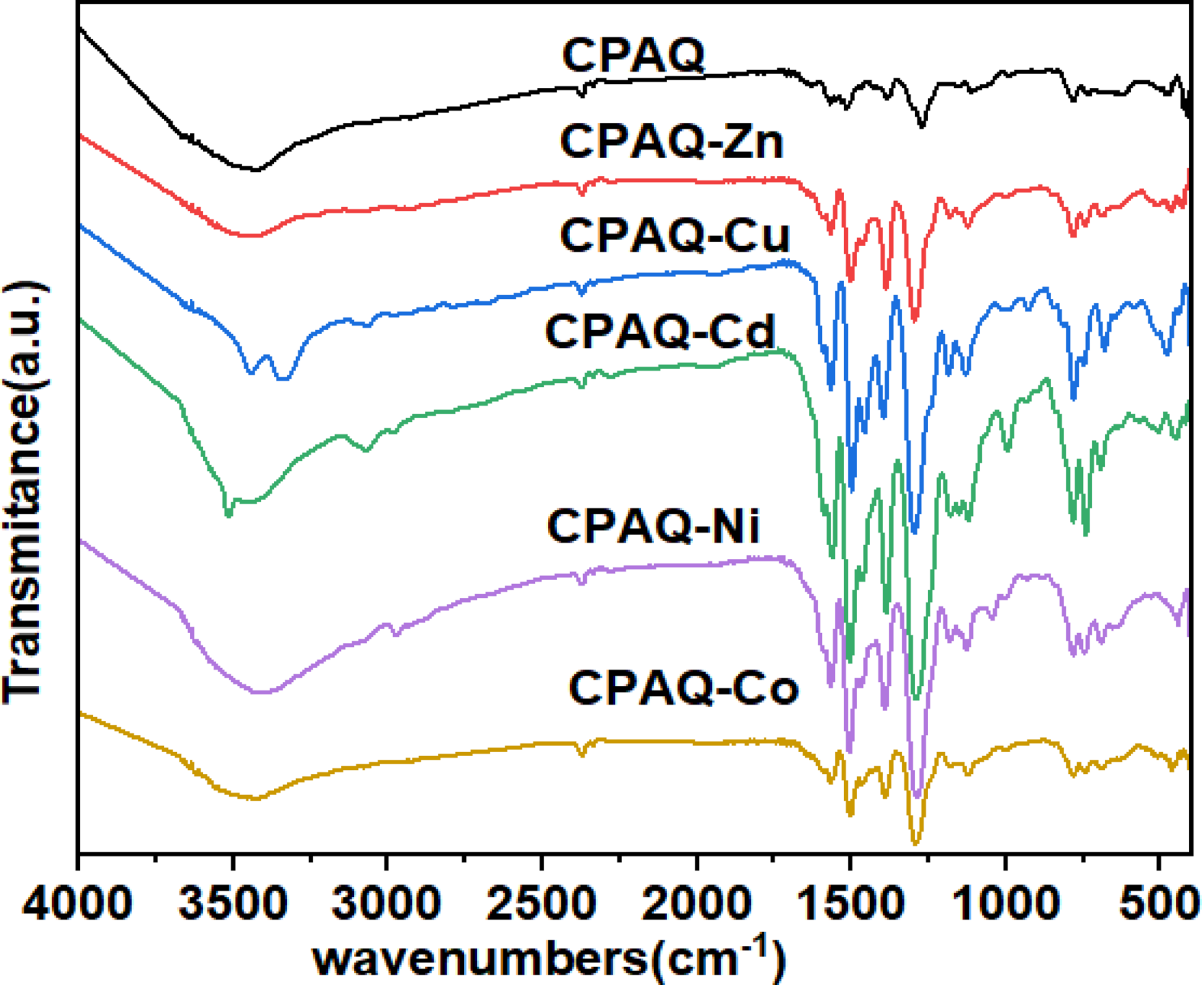
FTIR spectra of **CPAQ** ligand and its metallic derivatives.

By comparing the FTIR spectra of the metal complexes with that of the free ligand, **CPAQ**, the coordinating function groups in the ligand to the metal centers are detected. Coordinating function groups frequently exhibit positional and/or intensity fluctuations. Certain bands may also vanish as a result of chelation. Table 2 lists the most significant infrared bands of **CPAQ** and its complexes along with their assignment, and Fig. 3 displays the spectra of **CPAQ** and its metal complexes.

**Table 2:** The most important IR spectral bands (cm^-1^) of **CPAQ** ligand and its metallic complexes.

Comp	ν(OH)	ν(C=N)	ν(N=N)	ν(C–O)	ν(C–Cl)	ν(M–O)	ν(M–N)
CPAQ	3464	1570	1517	1272	783	–	–
CPAQ-Zn	3468	1570	1503	1293	782	507	431
CPAQ-Cu	3446	1570	1499	1294	785	512	439
CPAQ-Cd	3430	1570	1506	1285	785	528	453
CPAQ-Ni	3422	1568	1508	1297	784	450	442
CPAQ-Co	3464	1568	1506	1292	783	522	465

The band that appeared at 3464 cm^-1^ in the **CPAQ** spectra was assigned to the stretching vibrations of the OH group, according to the analysis of the results shown in Fig. 3 and Table 2. The stretching vibration of the C=N and N=N groups was identified as the cause of the bands that appeared at 1570 and 1517 cm^−1^, respectively^37^, whereas the bands appearing at 1272 and 783 cm^−1^ assigned to ν(C–O) and ν(C–Cl), successively^55–57^.

In the spectra of complexes, **CPAQ-Zn**, **CPAQ-Cu**, **CPAQ-Cd**, **CPAQ-Ni** and **CPAQ-Co**, the large shift in the positions of the two bands that match to N=N and C–O groups in contrast to their positions in the free ligand assured their ligation to the metal ion centers incorporated in complex structures. The first band (i.e. N=N) appeared in the range of 1499–1508 cm^−1^ affording a shift by 7–16 cm^−1^ to lower wavenumbers in comparison to the uncomplexed ligand. The second band appeared in the range of 1285–1294 cm^−1^ with a shift of 13–22 cm^−1^ to a higher value. However, the complexes’ spectra showed the bands for v(C=N) and ν(C–Cl) in the ranges of 1568–1570 and 782–785 cm^−1^, successively, with little or no shift in location relative to their parent ligand. Consequently, in complexes, these two bands did not coordinate with the metal centers and remained free. In the spectra of all metal complexes, a non-ligand band appearing in the ranges 507–537 and 431–465 cm^−1^ is attributed to v(M–O) and v(M–N), successively, which provides more evidence of the participation of the oxygen and nitrogen atoms in complex formation. The bands found in all complexes’ spectra between 3422 and 3468 cm^−1^ are attributed to the lattice and coordinated water molecules’ ν(OH) that are a part of the complexes’ structures^54^.

#### ^1^H-NMR spectra

**Fig. 4:**
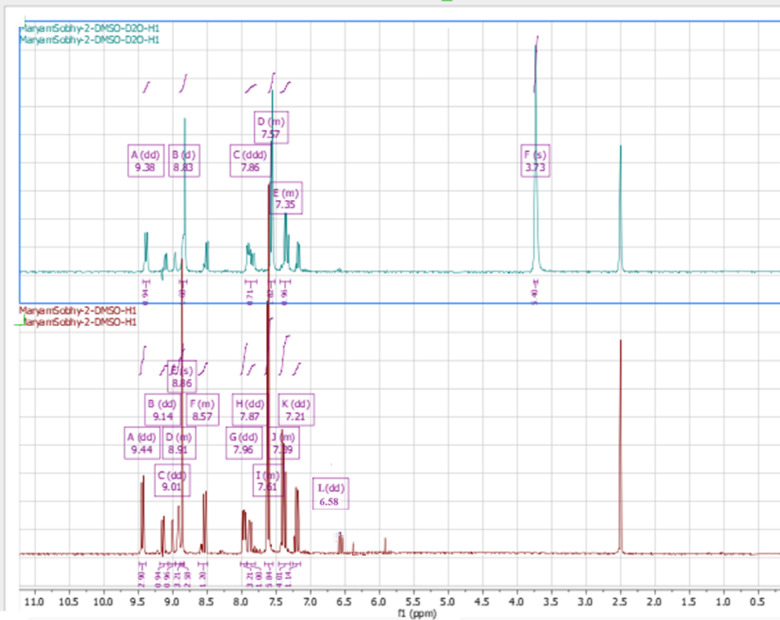
1H-NMR spectra of **CPAQ** ligand in d6-DMSO (down) and D_2_O (up).

^1^H-NMR spectrum of the ligand **CPAQ** was recorded in d^6^-DMSO and D_2_O using tetramethylsilane (TMS) as internal standard (Fig. 4). In DMSO, the ligand spectrum displayed four resonances at 9.44, 9.01, 8.86, and 7.61 ppm assigned to hydroxyl quinolone ring protons. The two resonances appearing at 7.39 and 7.21 ppm are assigned to the dichloro aromatic ring protons. Such signals still appear in the ligand spectrum upon the addition of D_2_O to the measurement solution. The singlet appearing at 5.87 ppm attributed to OH proton that completely vanished on the spectra measured in D_2_O. Another singlet signal appeared at 6.38 ppm which also completely disappeared with the addition of D_2_O. The appearance of such signal backs the existence of the ligand **CPAQ** in solution in 2 tautomeric forms that are suggested in Fig. 1^56^. The appearance of the signals at 9.14, 8.91, 8.57, 7.96, 7.87 and 6.58 ppm that can be assigned to 7 aromatic protons in the second tautomer assure the tautomerism suggested for the ligand structure.

**Fig. 5:**
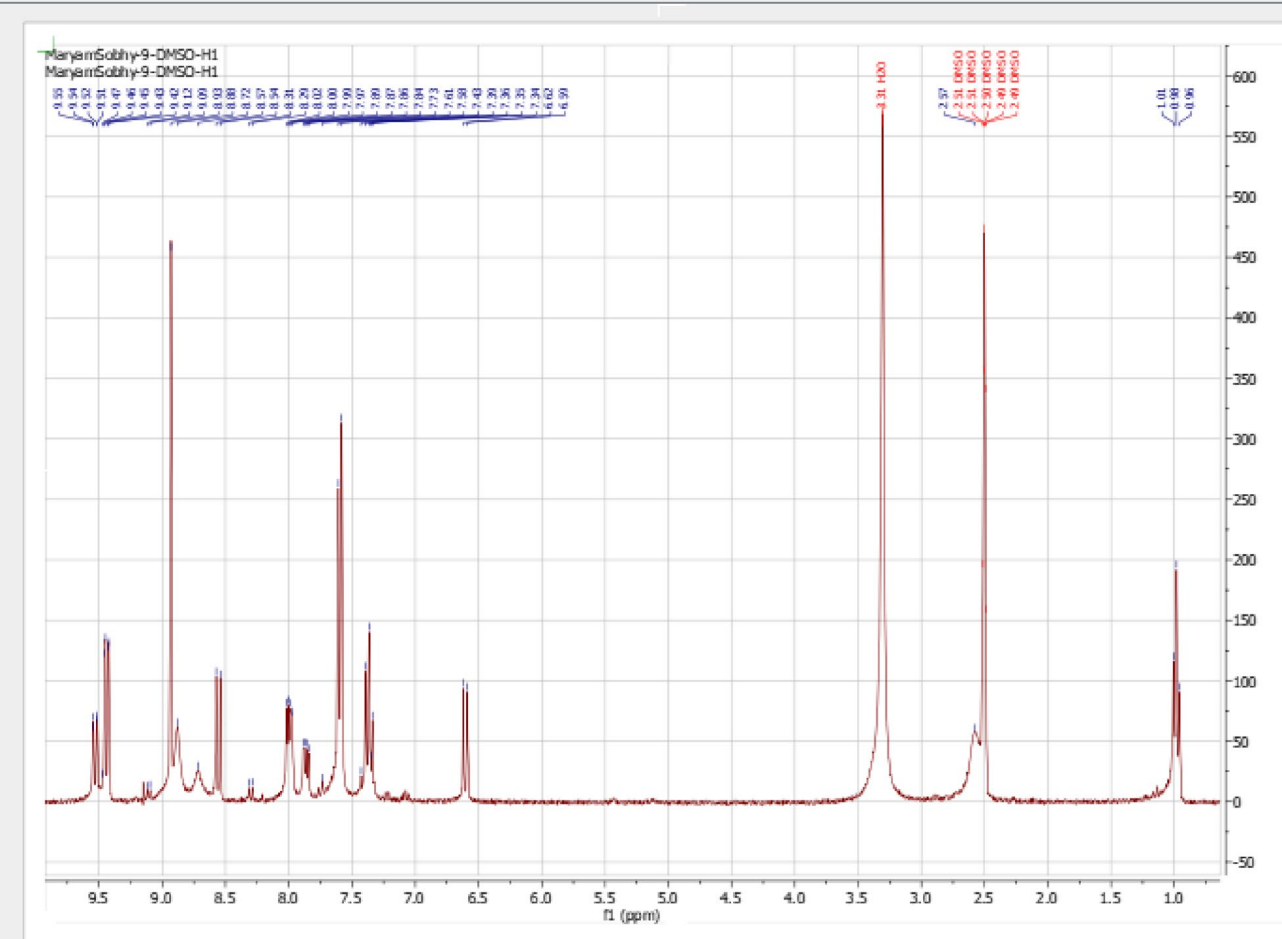
1H-NMR spectrum of **CPAQ-Cd** in d6-DMSO.

Additionally, the ^1^H-NMR spectra of **CPAQ-Zn** and **CPAQ-Cd** complexes (Fig. 5 and supplementary file) can be considered a perfect guide to assign the mode of binding of the ligand to the metal centre. The most important feature that is concluded from the precise investigation of the NMR spectra of **CPAQ-Zn** and **CPAQ-Cd** complexes is the complete disappearance of the singlet signal appearing at 5.87 ppm assuring the involvement of the oxygen atom of the OH group in complex formation through proton displacement.

#### Thermal gravimetric analytical results

**Fig. 6:**
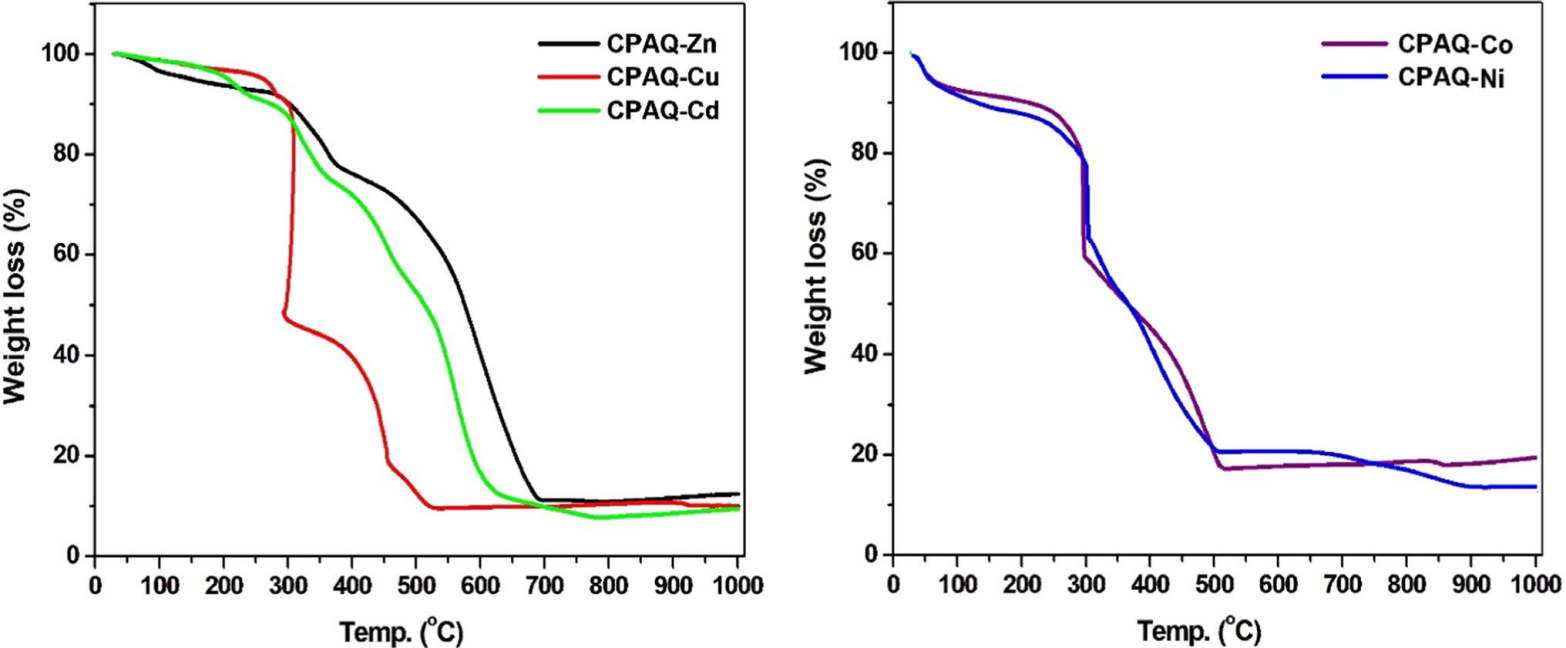
TG thermograms of metal complexes.

Thermal analysis is one of the most useful methods used to forecast the molecular structure and stability of compounds by introducing crucial data about their thermal properties, phases of thermal degradation, kinds of intermediates, and residual products of thermal degradation^23^.Anionic groups bonded to the metal center, as well as the proportion and kind of water and/or organic solvent molecules, can also be identified. **CPAQ** complexes’ TG thermograms are shown in Fig. 6 and Table 3 shows the thermograms’ analytical results.

**Table 3:** Thermogravimetric analysis results for the interested metal complexes.

Compound (M. wt) Empirical formula	Temp range (°C)	Weight loss (%)		Assignment
		Calc	Found	
**CPAQ-Zn** (816.71) [Zn(L)_2_]·1.5H_2_O	25–97	3.30	3.22	Loss of 1.5H_2_O (lattice)
	97–253	4.34	4.13	Loss of 0.5Cl_2_
	253–377	15.60	15.27	Loss of 2NO_2_ + 0.5Cl_2_
	377–702	67.26	66.50	Further decomposition of ligand leaving ZnO
**CPAQ-Cu** (796.85) [Cu(L)_2_]·0.5H_2_O	37–90	1.13	1.28	Loss of 0.5H_2_O (lattice)
	90–281	7.78	7.25	Loss of 1NO_2_ + 0.5O_2_
	281–296	43.92	43.45	Loss of 2Cl_2_ + NO + 2C_6_H_3_ + N_2_
	296–457	29.51	29.43	Loss of C_9_H_4_NO + N_2_ + C_4_H_3_N
	457–530	7.66	7.27	Further decomposition of ligand leaving CuO residue
**CPAQ-Cd** (836.71) [Cd(L)_2_]	29–244	8.47	8.37	Loss of Cl_2_
	244–364	17.44	16.87	Loss of C_6_H_3_Cl_2_
	364–475	18.06	18.58	Loss of C_6_H_3_N_2_ + NO_2_
	475–626	42.94	43.70	Further decomposition of organic moiety leaving Cd residue
**CPAQ-Ni** (510.34) [NiLCl(H_2_O)_3_]	23–226	10.58	10.36	Loss of 3H_2_O (coordination)
	226–410	35.55	35.31	Loss of 0.5Cl_2_ (coordinated) + C_6_H_3_Cl_2_
	410–548	33.70	3352	Loss of C_9_H_4_N_2_O_2_
	548–990	5.48	6.14	Loss of N_2_ leaving NiO residue
**CPAQ-Co** (474.55) [CoLCl(H_2_O)]	30–110	3.79	4.07	Loss of 1H_2_O (coordination)
	110–371	36.27	35.91	Loss of C_9_H_4_N_2_O_2_ moiety
	371–553	41.60	42.21	Further decomposition of ligand leaving CoO + C

For the 5 investigated complexes, **CPAQ-Zn**, **CPAQ-Cu**, **CPAQ-Cd**, **CPAQ-Ni** and **CPAQ-Co,** the decomposition of the complexes took place in successive 3, 4 or 5 decomposition steps. **CPAQ-Co** decomposed within 3 stages, **CPAQ-Zn**, **CPAQ-Cd** & **CPAQ-Ni** decomposed in 4 steps and **CPAQ-Cu** in 5 steps**.**

According to an analysis of the five complexes’ TG thermograms, the two complexes, **CPAQ-Zn** and **CPAQ-Cu** underwent the removal of hydration water molecules in the first decomposition step in the temperature range 37–97 and 37–90 °C, respectively, with weight loss of 3.22 (calculated: 3.30) % and 1.28 (calculated: 1.13) %, respectively. The first decomposition step for the complexes **CPAQ-Ni** and **CPAQ-Co** was assigned to the removal of coordination water molecules where these stages appeared in the ranges 23–226 and 30–110 °C, respectively, with weight loss of 10.36 (calculated: 10.58) % and 4.07 (calculated: 3.79). For **CPAQ-Cd**, With a weight loss of 8.37 (calculated: 8.47) percent, the first stage of disintegration occurred in the temperature range of 29–244 °C and was attributed to the loss of Cl_2_ contained in the complex structure. The loss of coordinated chloride ions and the organic ligand’s breakdown occurred during the remaining phases of decomposition.

For **CPAQ-Zn** complex, the second, third and fourth decomposition steps appeared at 97–253, 253–377 and 377–702 °C with weight loss of 4.13 (calculated: 4.34) %, 15.27 (calculated: 15.60) % and 66.50 (calculated: 67.26) %, respectively.

For **CPAQ-Cu** complex, the remaining 4 steps of decomposition appeared within the ranges 90–281, 281–296, 296–457, 457–530 °C associated with weight loss of 7.25 (calculated: 7.68) %, 43.45 (calculated: 43.92) %, 29.43 (calculated: 29.51) % and 7.27 (calculated: 7.66) %, respectively.

The remaining 3 stages of **CPAQ-Cd** complex appeared in the ranges 244–364, 364–475, and 475–626 °C with weight loss percentage of 16.87 (calculated: 17.44) %, 18.58 (calculated: 18.06) % and 43.70 (calculated: 42.94) %, successively.

The remaining steps of decomposition for **CPAQ-Ni** complex appeared within the stages 226–410, 410–548 and 548–990 °C associated with weight loss of 35.31 (calculated: 35.55) %, 33.52 (calculated: 33.70) (calculated: 18.06) % and 4.14 (calculated: 5.48) %, successively. For **CPAQ-Co** complex, the remaining two decomposition steps appeared in the ranges 110–371 and 371–553 °C with weight loss percentages of 35.91 (calculated: 36.27) % and 42.21 (calculated: 41.60) %, respectively. All the complexes left whether the metal or metal oxide residues as decomposition products at the end of decomposition reactions as depicted in Table 3.

#### Electronic absorption and magnetic moment measurements

The electronic absorption spectra of all chelates were studied using Nujol mull technique to get insight into the geometrical arrangement around the metal centre in the metal chelates. The spectra were recorded within the range 200–800 nm.

In **CPAQ-Cu** complex’s spectrum, the band that is located nearly at 423 nm is attributed to the charge transfer transition. The broad band appearing at about 655 nm is assigned to the d-d electronic transition of the type ^2^T_2_ → ^2^E reported for Cu complexes with distorted tetrahedral geometry^58–60^.

The electronic absorption spectrum of Co^2+^ chelate, **CPAQ-Co**, showed two bands at 552 and 426 nm which may be assigned to ^4^A_2_ → ^4^T_1_ (F) and ^4^A_2_ → ^4^T_1_ (p) transitions, respectively, assuming the tetrahedral geometry around the Co^2+^ ion^61,62^.

The electronic absorption spectrum of the Ni^2+^ chelate, **CPAQ-Ni,** exhibited two bands at 680 and 540 nm corresponding to ^3^A_2g_ → ^3^T_1g_(F) and ^3^A_2g_ → ^3^T_1g_ (P) transitions, respectively, that shows the octahedral geometry around Ni^2+^ ion^62,63^.

For the two complexes **CPAQ-Zn** and **CPAQ-Cd** with d^10^ electronic structures, the non-ligand bands appearing at 510 and 520 nm, respectively, assigned to the charge transfer transition. Furthermore, as was to be expected, their electrical spectra provided no useful insights into stereochemistry^63^.

The calculated values of the magnetic moment for Co^2+^, Ni^2+^ and Cu^2+^ chelates are 4.3, 3.6 and 2.1 B.M., respectively. These values are in agreement with the proposed geometrical structures. As a result of spin–orbit coupling, the magnetic moment values are elevated above the theoretical spin-only value.

### Theoretical studies

#### Geometrical structure

**Fig. 7:**
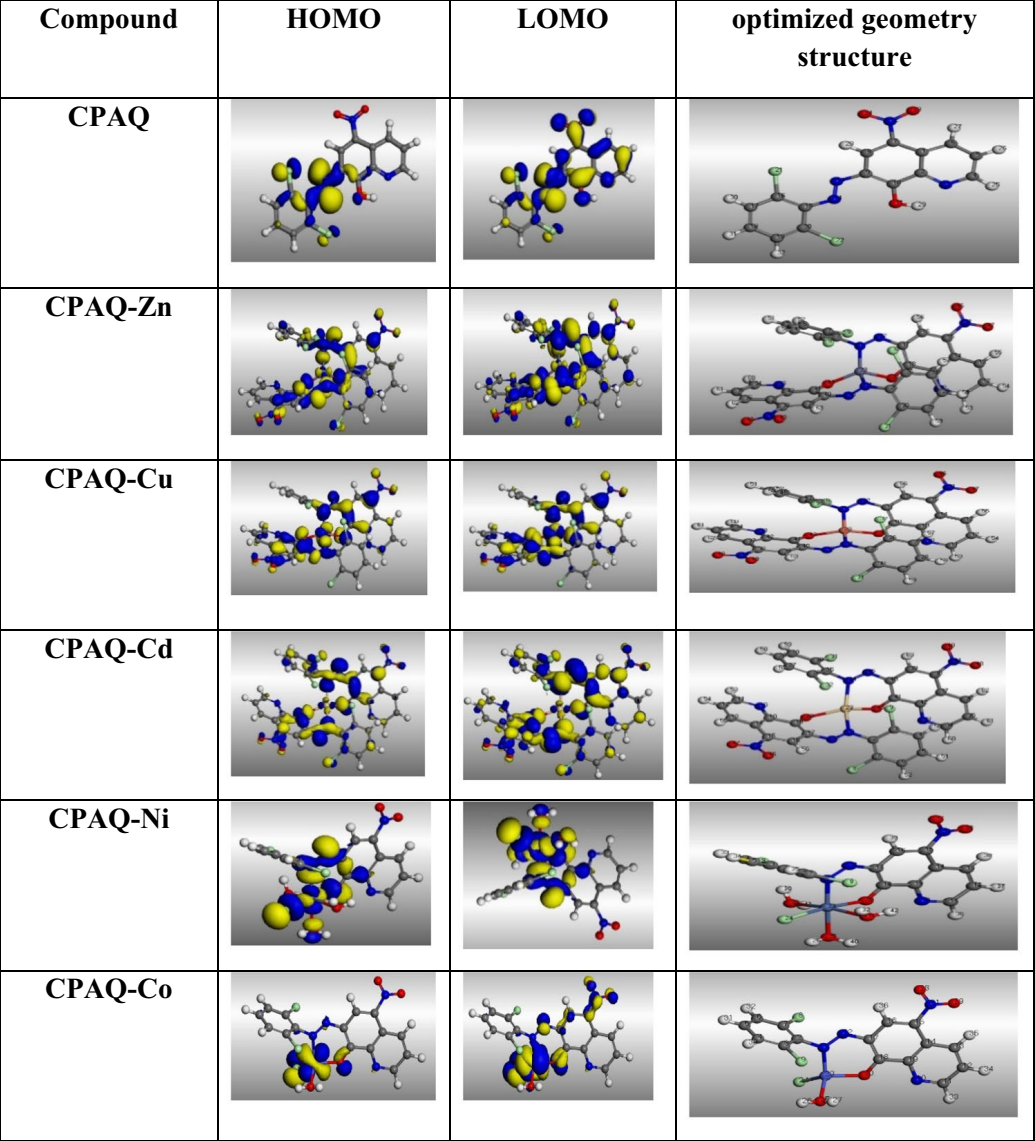
HOMO, LUMO, and optimized structures of **CPAQ** ligand and its metal complexes based on DMOL^3^ module adopted in the Materials Studi, 2017.

In order to better understand the geometrical structures of the five complexes under investigation that is, whether they are square planar, tetrahedral, or octahedral in geometry, molecular modeling studies were conducted using the Material Studio program because our attempts to obtain a single crystal of the metal complexes have so far failed. The optimal structures of the synthesized compounds’ bond lengths and angles are depicted in the supplementary file with a highlight on the most important bond lengths and angles. The optimized structures are shown in Fig. 7. The ligand and its complexes’ acquired bond lengths and angles were precisely examined, leading to the following conclusions:

1. From structures shown in Fig. 7, it is clear that Zn, Cu and Cd centers are surrounded by two N and two O atoms from two ligand molecules whereas Co is surrounded by one N and one O atom from one ligand molecule in addition to one O atom of coordinated water molecule and one coordinated Cl atom affording 4 coordination geometries for the 4 complexes. The value of bond angles for **CPAQ-Zn, CPAQ-Cu, CPAQ-Cd** and **CPAQ-Co** complexes supported tetrahedral geometry around the metal centers with distortion. From the ideal tetrahedral geometry, the minimum and maximum coordination angles around the core metal atom indicate the degree of distortion. The minimum angles are 94.72, 98.61, 100,28 and 95.64° and the maximum angles are 120.04, 127.04, 123.70 and 122.45° for **CPAQ-Co, CPAQ-Cu(II), CPAQ-Zn(II)** and **CPAQ-Cd(II)** complexes, respectively.
2. For **CPAQ-Ni** complex, the Ni center had a hexa-coordination structure with N and O of one ligand molecule, three oxygen atoms of three coordinated water molecules and one coordinated Cl atom resulting in a slightly distorted octahedral geometry around the Ni centre. The degree of distortion can be rationalized from the values of the cis and trans angles which have ideal values of 90 and 180°, respectively. For **CPAQ-Ni** complex, the minimum and maximum measure of the trans angles are 177.3° and 179.40^°^, respectively, whereas the minimum and maximum measure of the cis angles are 87.39 and 93.14^°^, respectively.
3. Coordination of the ligand atoms to the metal centers in the metal complexes results in a discernible alteration in bond length, leading to either shortening or elongation upon complex formation. This is obvious for the azo (N=N) and C–O bonds, which were found to be 1.4117 and 1.4513 Å, respectively, in the ligand **CPAQ.** Such bonds underwent obvious elongation in all metal complexes except for **CPAQ-Co** in which the two bonds were shortened equalled with the free ligand. Such an alteration in these two bond lengths can be taken as evidence for their ligation to the metal center through the nitrogen and oxygen atoms.
4. Additionally, the length of two bonds M–N and M–O (of deprotonated hydroxyl oxygen) in the five metal complexes can be arranged as follow: Ni–N > Cd‐N > Cu‐N > Zn‐N > Co–N and Cd‐O > Ni–O > Cu‐O > Zn‐O > Co–O showing the strongest bonds for **CPAQ-Co** complex and hence reflect its coordination stability^50,64^. Such suggested increased stability of **CPAQ-Co** complex over the remaining complexes is also supported by the decrease in the length of N=N and C–O bonds in comparison to their lengths in the free ligand in an opposite behavior to the remaining complexes.
5. All bond lengths and angles are within the normal values ^65^.

#### Frontier molecular orbitals and chemical reactivity

The CPAQ’s frontier molecular orbitals are its lowest unoccupied molecular orbital (LUMO) and its highest occupied molecular orbital (HOMO) and its complexes are represented in Fig. 7 The values of the electronic structure parameters (E_HOMO_, E_LUMO_, E_Gap_) are collected in Table 4 in addition to the various molecular parameters based on E_HOMO_ and E_LUMO_ values (electron affinity (EA) and ionization potential (IP)) which are calculated as the following Eq. ^50^.

$$\begin{aligned}&\Delta {\text{E}} = {\text{ E}}_{{{\text{LUMO}}}} - {\text{ E}}_{{{\text{HOMO}}}}\\&{\text{IP}} = \, - {\text{E}}_{{{\text{HOMO}}}}\\& {\text{EA}} = - {\text{E}}_{{{\text{LUMO}}}} \end{aligned}$$

**Table 4:** The values of the electronic structure parameters of the ligand and its complexes.

Compound	HOMO	LOMO	ΔE	IP	EA
CPAQ	− 0.1947	− 0.1379	0.0568	0.1947	0.1379
CPAQ-Zn	− 0.2173	− 0.1613	0.056	0.2173	0.1613
CPAQ-Cu	− 0.2209	− 0.184	0.0369	0.2209	0.184
CPAQ-Cd	− 0.2123	− 0.148	0.0643	0.2123	0.148
CPAQ-Ni	− 0.1908	− 0.144	0.0468	0.1908	0.144
CPAQ-Co	− 0.2007	− 0.1657	0.035	0.2007	0.1657

The ability of a molecule to donate electrons is known to be correlated with its HOMO, and a higher E_HOMO_ value (i.e., a lower ionization potential) indicates a better electron-donating ability. On the other hand, LUMO is linked to a molecule’s capacity to receive electrons; a smaller E_LUMO_ value and a higher electron affinity value indicate a strong capacity to accept electrons. To assess the reactivity of chemicals, the HOMO–LUMO gap (ΔE) is also a crucial metric. Generally, the *−ve* values of *E*_HOMO_ and *E*_LUMO_ imply the high stability of isolated compounds^66,67^. Some researchers also think that a low Δ*E* indicates that the molecule is more reactive^68,69^.

However, other scientists note that the trend of a compound’s reactivity cannot be directly predicted by analyzing its molecular electrical structure. In fact, the factors that affect the reactivity are complicated^70^.

The data in Table 4 shows the reactivity of synthesized compounds following the order **CPAQ-Co > CPAQ-Cu > CPAQ-Ni > CPAQ-Zn > CPAQ > CPAQ-Cd**, according to the energy gap values, in which the order agrees well with the experimental outcome due to that the ΔE maybe is the affecting factor.

**Fig. 8:**
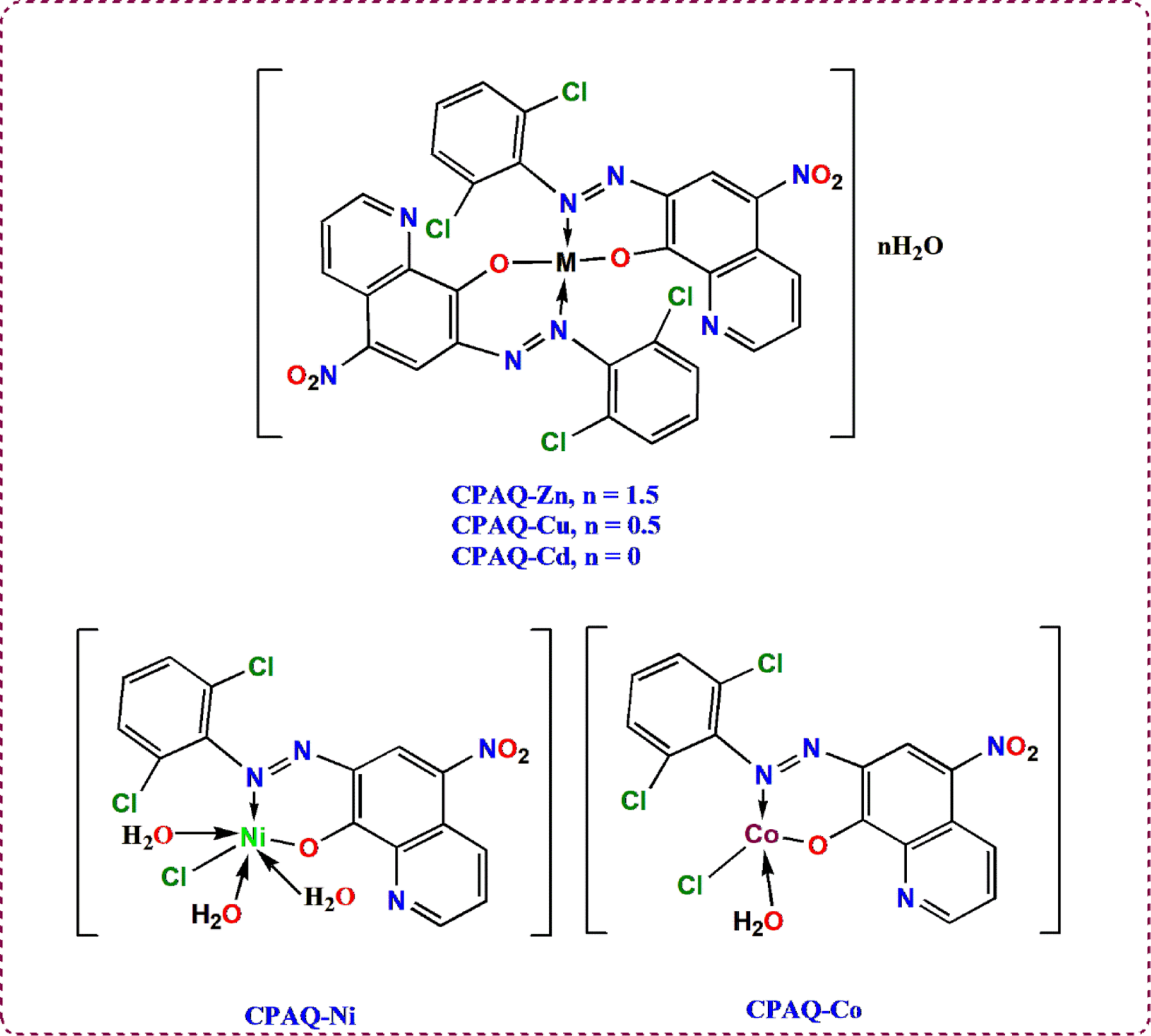
Proposed structures of the metal chelates.

As a conclusion of all the previous results, the structures of the metal complexes **CPAQ-Zn, CPAQ-Cu, CPAQ-Cd, CPAQ-Ni** and **CPAQ-Co** are sketched in Fig. 8.

### In‑silico molecular docking

**Fig. 9:**
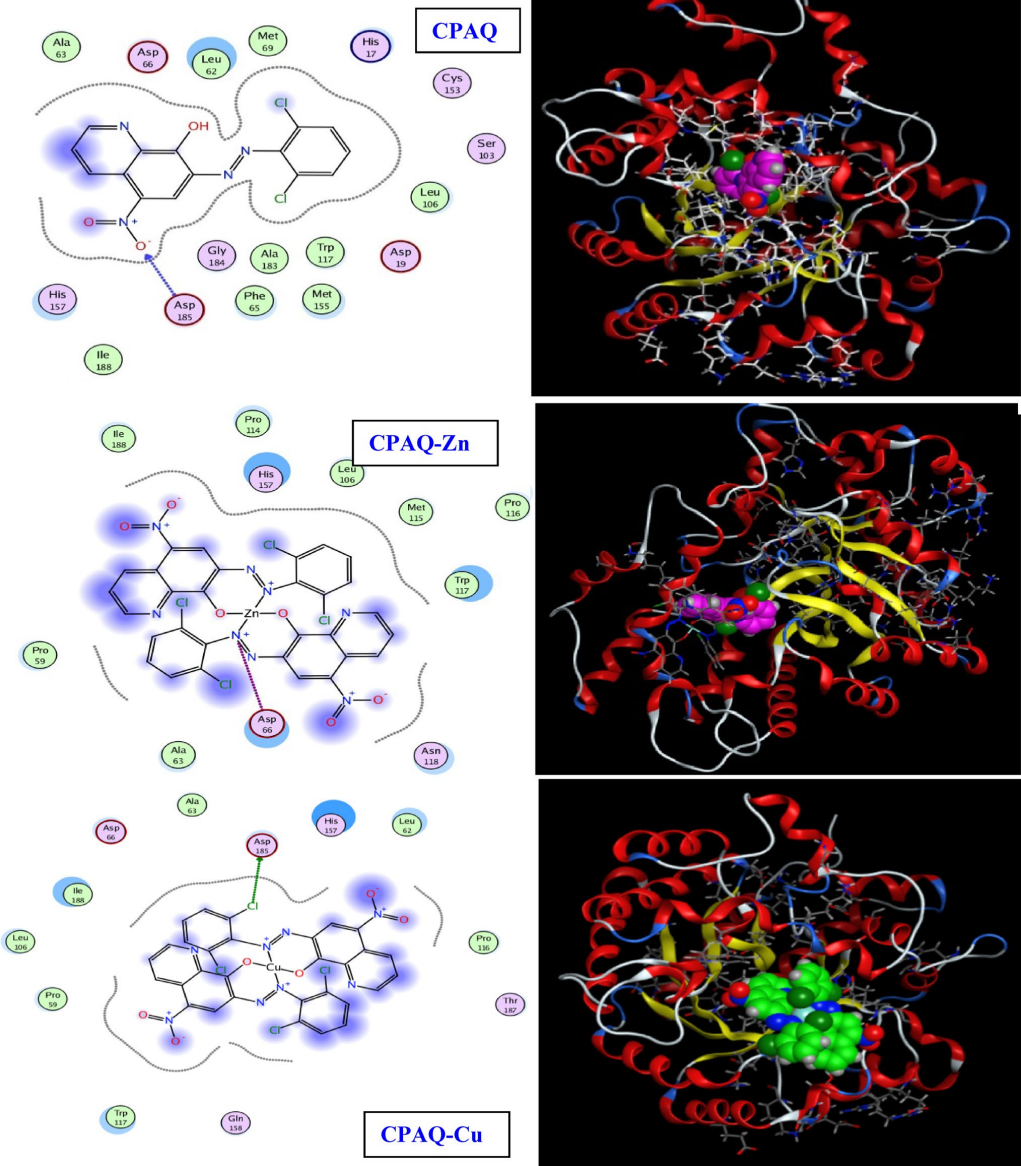
2D and 3D view of binding interaction of **CPAQ, CPAQ-Zn** and **CPAQ-Cu** with the *Mus musculus* ADA enzyme structure (PDB ID: 1a4m) using MOE-Dock 2014 software.

**Fig. 10:**
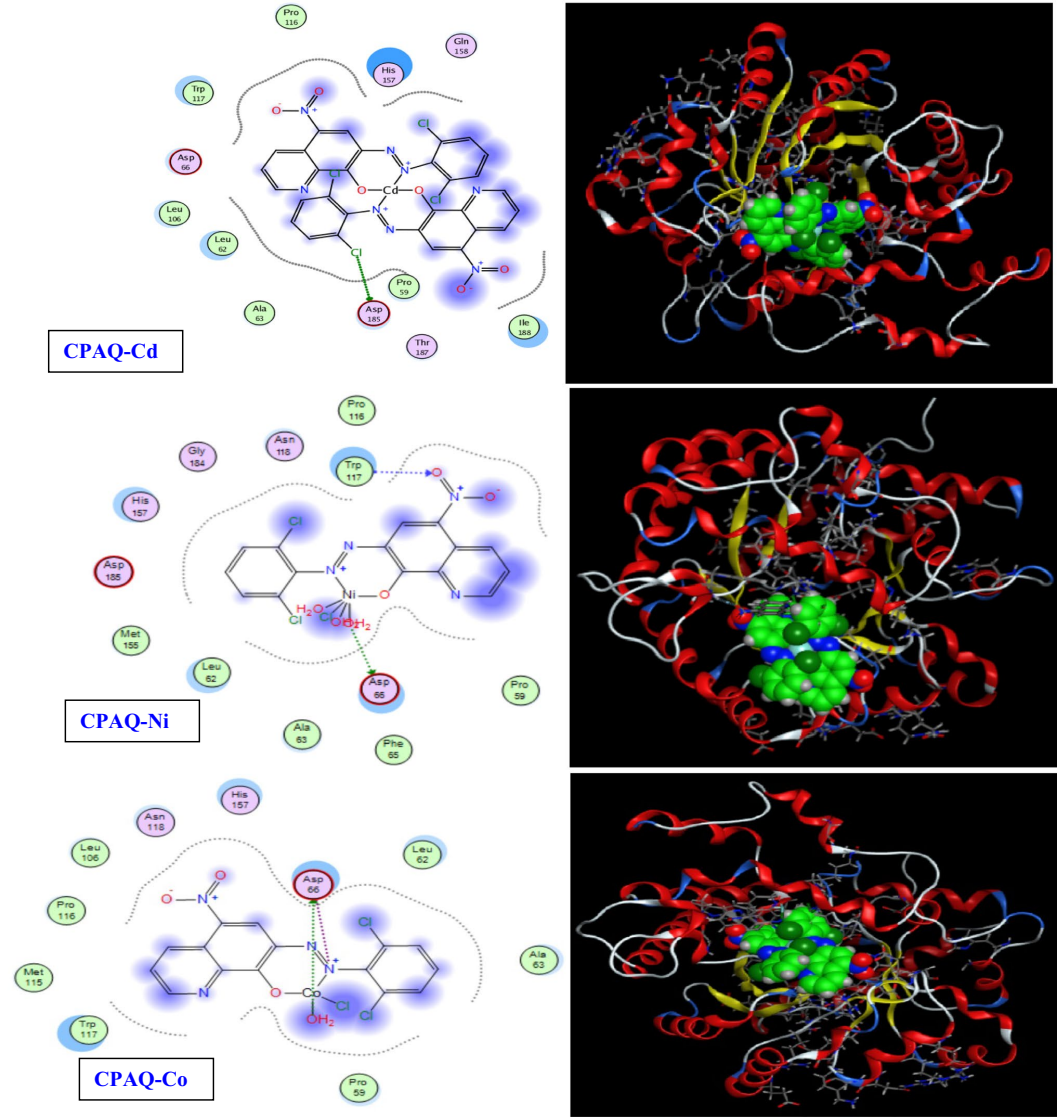
2D and 3D view of binding interaction of **CPAQ-Cd, CPAQ-Ni** and **CPAQ-Co** with the *Mus musculus* ADA enzyme structure (PDB ID: 1a4m) using MOE-Dock 2014 software.

The interactions of the target compounds (the ligand **CPAQ** and complexes **CPAQ-Zn, CPAQ-Cu, CPAQ-Cd, CPAQ-Ni** and **CPAQ-Co)** with the *Mus musculus* ADA enzyme structure (PDB ID: 1a4m) that is obtained from protein data bank (PDB) was evaluated theoretically applying molecular docking studies. The results showing the molecular interactions of the interested compounds with 1a4m are shown in Table 5 and represented in Figs. 9 and 10. From the results in Table 5 it is well obvious that the strongest binding with 1a4m is observed for **CPAQ-Co** complex with a docking score of 6.4693 kcal/mol (−ve value). For this complex, 4 types of interactions were observed between both the azo nitrogen atom and oxygen atom of the coordinated water molecule with the amino acid moiety ASP 66 with bond type interaction of H donor and ionic. The following strongest interaction was observed by **CPAQ-Zn** complex affording a docking score of 5.7307 kcal/mol (−ve value). The interaction of this complex was ionic interaction observed between the azo nitrogen of the metal complex with the amino acid ASP 66. Both **CPAQ-Cu** and **CPAQ-Cd** afforded interaction with 1a4m with docking score of − 5.6148 and − 5.6052, respectively, with H donor interactions between chloride atom of 2,6-dichlorobenzene ring and ASP 185 moiety. The least interaction was observed by free ligand **CPAQ** followed by the **CPAQ-Ni** with docking scores of − 5.1549 and − 5.3416 kcal/mol, respectively. The ligand interaction was observed between an oxygen atom of NO_2_ group and ASP 185 amino acid; the type of interaction is H-acceptor interaction. For **CPAQ-Ni** complex, two types of interactions were observed two oxygen atoms of two of the coordinated water molecules with ASP 66 amino acid with H-donor interaction type. Thus, the order of strength of interaction between 1a4m and the studied compounds follows the order **CPAQ-Co** > **CPAQ-Zn** > **CPAQ-Cu** ≈ **CPAQ-Cd** > **CPAQ-Ni** > **CPAQ**. It is also notable that all the lengths of bonds for every interaction, except for **CPAQ-Zn**, are less than 3.5 Å affording a true docking track^54,67^.

**Table 5:** The results of the molecular interactions of the interested compounds with 1a4m.

Compound	Ligand moiety	Receptor site	Type of interaction	Distance (A^o^)	E (kcal/mol)	Docking score (kcal/mol)
CPAQ	O 17	N ASP 185 (A)	H-acceptor	3.10	− 2.2	− 5.1549
CPAQ-Zn	N 30	OD2 ASP 66 (A)	Ionic	3.82	− 0.9	− 5.7307
CPAQ-Cu	CL 41	OD2 ASP 185(A)	H-donor	3.54	− 0.4	− 5.6148
CPAQ-Cd	CL 12	OD2 ASP 185(A)	H-donor	3.53	− 0.4	− 5.6052
CPAQ-Ni	O 32	OD1 ASP 66 (A)	H-donor	2.74	− 3.3	− 4.1885
	O 32	OD2 ASP 66 (A)	H-donor	2.88	− 11.6	
	O 42	N TRP 117 (A)	H-acceptor	2.98	− 2.0	
	O 32	OD1 ASP 66 (A)	Ionic	2.74	− 6.4	
	O 32	OD2 ASP 66 (A)	Ionic	2.88	− 5.3	
CPAQ-Co	O 32	OD2 ASP 66 (A)	H-donor	2.68	− 2.5	− 6.4693
	N 1	OD1 ASP 66 (A)	Ionic	3.19	− 3.3	
	N 1	OD2 ASP 66 (A)	Ionic	3.21	− 3.2	
	O 32	OD2 ASP 66 (A)	Ionic	2.68	− 7.0

### Inhibition of ADA enzyme by metal complexes

**Fig. 11:**
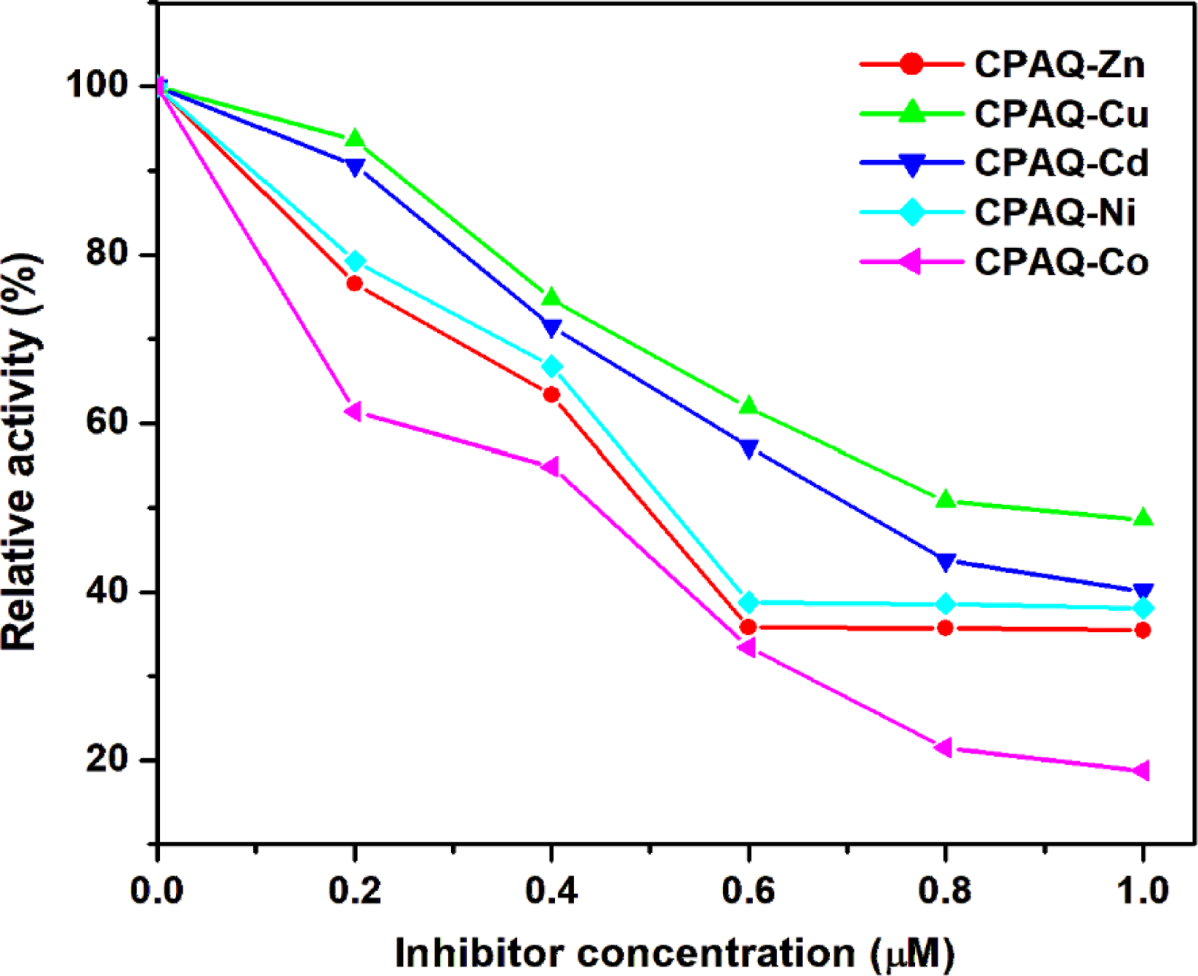
Inhibition of ADA enzyme activity by various concentrations of metal complexes.

ADA plays a crucial role in purine metabolism and its elevated activity is linked to various pathological conditions, including inflammation and autoimmune diseases. The observed inhibitory effects of metal complexes under interest on ADA activity suggest potential regulatory mechanisms that may influence joint health and inflammatory responses. The inhibitory activities of the five tested metal complexes were compared based on the data summarized in Table 6 and visualized in Fig. 11. Enzymatic kinetic studies provided a deeper understanding of how adenosine deaminase (ADA) behaves under varying substrate concentrations. The Lineweaver–Burk plots derived from the kinetic data revealed that ADA functions efficiently at lower substrate concentrations, with its activity gradually reaching a plateau as the substrate becomes saturated. Among the tested complexes, the **CPAQ-Co** complex stood out as the most potent inhibitor. As shown prominently in Fig. 11, this complex reduced ADA activity significantly, bringing it down to 18.74% of its original activity at a low concentration of 1 µM. This finding underscores the therapeutic potential of **CPAQ-Co** in regulating purine metabolism, particularly in conditions like joint diseases where ADA activity plays a crucial role. This figure also represents the reactivity to follow the order **CPAQ-Co > CPAQ-Zn > CPAQ-Ni > CPAQ-Cd > CPAQ-Cu**. The IC_50_ values follow the same order where the tested compounds showed IC_50_ values within the range 0.445–0.875 µM showing that **CPAQ-Co** complex is the most active inhibitor and **CPAQ-Cu** is the least active one.

**Table 6:** The values of half maximal inhibitory concentration (IC_50_, µM) and inhibition constant (Ki, µM) for inhibition of ADA enzyme activity by various concentrations of metal complexes.

Inhibitor	IC_50_ (µM)	K_i_ (µM)
CPAQ-Zn	0.497	0.058
CPAQ-Cu	0.875	0.23
CPAQ-Cd	0.707	0.115
CPAQ-Ni	0.520	0.088
CPAQ-Co	0.445	0.028

**Fig. 12:**
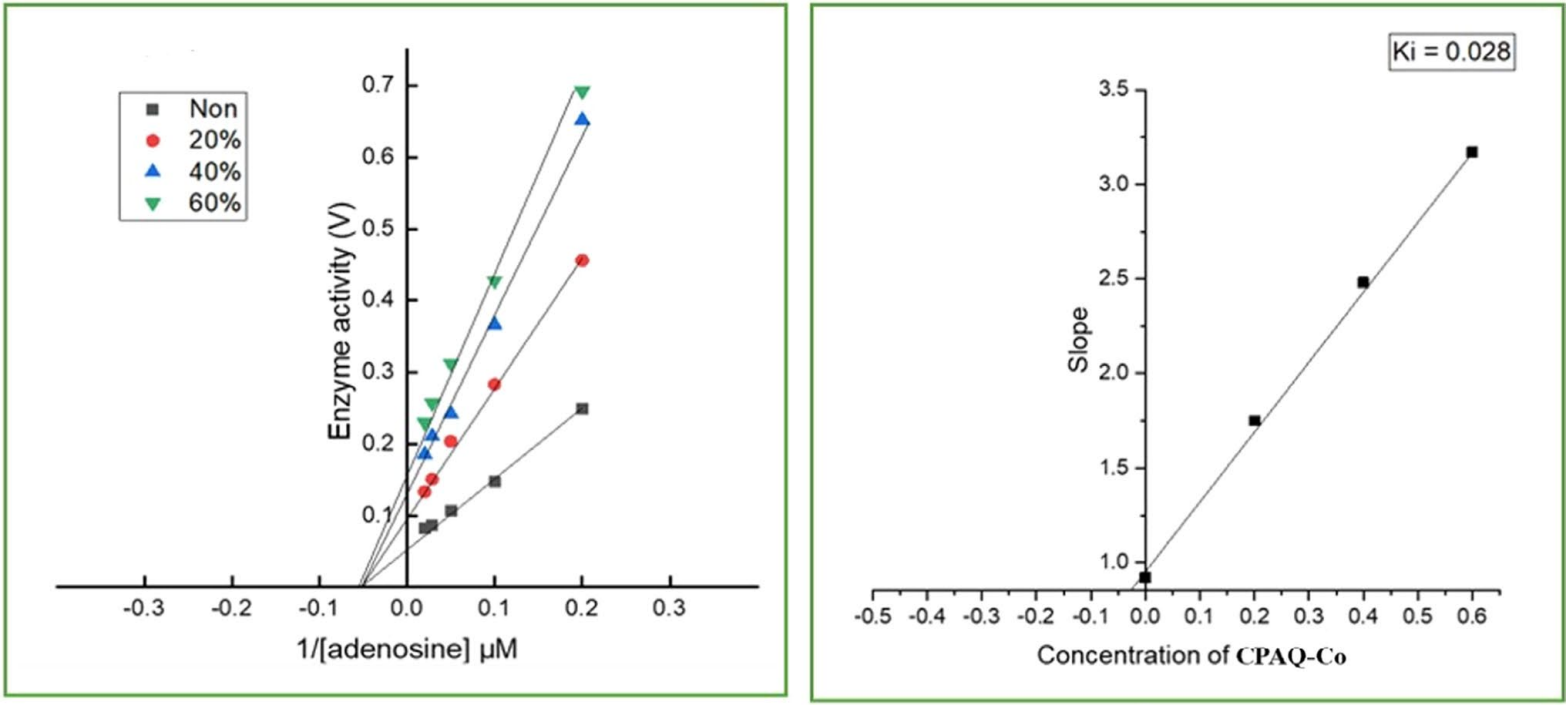
Lineweaver Burk plot and the inhibition constant (Ki) of ADA by **CPAQ-Co** complex.

According to the Lineweaver Burk plot, as the Vmax rose while Km remained constant, the metal complexes at concentrations of 0, 0.2, 0.4, 0.6, 0.8 and 0.1 µM/L demonstrated inhibition (Fig. 12). The inhibition type was hence noncompetitive. The Ki values were discovered to fall between 0.028 and 0.23 µM. The **CPAQ-Co** complex’s inhibition constant (Ki) of ADA and the Lineweaver–Burk plot are displayed in Fig. 12 as an example.

The promising results obtained recommend further research to explore the role of ADA inhibition in joint diseases and to evaluate the therapeutic potential of specific metal ions in controlling enzyme activity, which could pave the way for novel treatments targeting purine metabolism in joint-related disorders.

## Conclusion

Tetrahedral Zn(II), Cu(II), Cd(II) and Co(II) complexes and the octahedral Ni(II) complex of the newly synthesized azo dye ligand named 7-((2,6-Dichloro-phenylazo)-5-nitro-quinoline-8-ol (**CPAQ**) have been isolated. In all complexes, the ligand coordinates to the metal centres by the azo group nitrogen and C–O oxygen, as concluded from the results of FTIR. So, the ligand acted as monobasic bidentate. DFT simulations with the basis set GGA-PBE utilizing the DMOL3 module were used for structure optimization. The Co(II) complex was found to be the most reactive, according to HOMO and LOMO calculations. To assess the ability of the synthesized complexes to inhibit adenosine deaminase enzyme (ADA), molecular docking experiments have been performed through the study of the interactions between the ligand and complexes with the Mus musculus ADA enzyme structure (PDB ID: 1a4m). Additionally, the inhibitory effect of the synthesized compounds on adenosine deaminase enzyme (ADA) activity was tested in-vitro. The obtained results showed that Co(II) complex reduced ADA activity significantly, decreasing it to 18.74% of its original activity at a concentration of 1 µM. This finding underscores the therapeutic potential of **CPAQ-Co** in regulating purine metabolism, particularly in conditions like joint diseases where ADA activity plays a crucial role.

## Electronic supplementary material

Below is the link to the electronic supplementary material.

Supplementary Material 1

## Data Availability

All data generated or analysed during this study are included in this published article [and its supplementary information files].

## References

[1] Eltaboni, F., Bader, N., El-Kailany, R., Elsharif, N. & Ahmida, A. Dyes: A comprehensive review. *J. Chem. Rev.* **4**, 313–330 (2022).

[2] Sahar, Y. J., Mohammed, H. & Al-Abady, Z. N. Synthesis and characterization of new metal complexes containing azo-indole moiety and anti-leukemia human (HL-60) study of its palladium (II) complex. *Results Chem.* **5**, 100847 (2023).

[3] Rashidnejad, H. et al. A comprehensive spectroscopic, solvatochromic and photochemical analysis of 5-hydroxyquinoline and 8-hydroxyquinoline mono-azo dyes. *J. Mol. Struct.* **1223**, 129323 (2021).

[4] Park, H., Kim, E.-R., Kim, D. J. & Lee, H. Synthesis of metal-azo dyes and their optical and thermal properties as recording materials for DVD-R. *Bull. Chem. Soc. Jpn.* **75**, 2067–2070 (2002).

[5] Gao, T., Xue, Y., Zhang, Z. & Que, W. Multi-wavelength optical data processing and recording based on azo-dyes doped organic-inorganic hybrid film. *Opt. Express* **26**, 4309–4317 (2018). 29475282 10.1364/OE.26.004309

[6] Anger, E. & Fletcher, S. P. Simple azo dyes provide access to versatile chiroptical switches. *Eur. J. Org. Chem.* **2015**, 3651–3655 (2015).

[7] Samanta, S. et al. Photoswitching azo compounds in vivo with red LightClick to copy article link. *J. Am. Chem. Soc.* **135**, 9777–9784 (2013). 23750583 10.1021/ja402220t

[8] Soreño, Z. V., Puguan, J. M. C. & Kim, H. Thermochromic transition analysis of elastomer prepared from azo dye-siloxane blend. *Mater. Chem. Phys.* **240**, 122297 (2020).

[9] El-Ghamry, H. A., Alharbi, B. K., Takroni, K. M. & Khedr, A. M. A series of nanosized Cu(II) complexes based on sulfonamide azo dye ligands: An insight into complexes molecular structures, antimicrobial, antitumor and catalytic performance for oxidative dimerization of 2-aminophenol. *Appl. Organomet. Chem.* **37**, e6978 (2023).

[10] El-Ghamry, H. A., Alkurbi, A. A., Alhasani, M. A., Takroni, K. M. & Khedr, A. M. A series of nanosized Cu(II) complexes based on sulfonamide azo dye ligands: An insight into complexes molecular structures, antimicrobial, antitumor and catalytic performance for oxidative dimerization of 2-aminophenol. *Arab. J. Chem.* **16**, 104916 (2023).

[11] Ferreira, G. R. et al. Supramolecular compounds of azo dyes derived from 1-phenylazo-2-naphthol and their nickel and copper complexes. *Supramol. Chem.* **27**, 13–20 (2014).

[12] Schab-Balcerzak, E. et al. Comparative studies of polyimides with covalently bonded azo-dyes with their supramolecular analoges: Thermo-optical and photoinduced properties. *Opt. Mater. Amst.* **36**, 892–902 (2014).

[13] Matharu, A. S., Jeeva, S., Huddleston, P. R. & Ramanujam, P. S. Synthesis and optical storage properties of a thiophene-based holographic recording medium. *J. Mater. Chem.* **17**, 4477–4482 (2007).

[14] Dinçalp, H., Toker, F., Durucasu, İ, Avcıbaşı, N. & Icli, S. New thiophene-based azo ligands containing azo methine group in the main chain for the determination of copper(II) ions *Dye*. *Pigment* **75**, 11–24 (2007).

[15] Omar, A. Z., El-Rahman, M. A., El-Sadany, S. K., Hamed, E. A. & El-Atawy, M. A. Synthesis of novel bisazo disperse dyes: Spectroscopic characterization, DFT study and dyeing of polyester. *Dye. Pigment.* **196**, 109831 (2021).

[16] Almáši, M., Vilková, M. & Bednarčík, J. Synthesis, characterization and spectral properties of novel azo-azomethine-tetracarboxylic Schiff base ligand and its Co (II), Ni (II), Cu (II) and Pd (II) complexes. *Inorg. Chim. Acta* **515**, 120064 (2021).

[17] Abd El-Lateef, H. M. et al. Design, synthesis of new mixed azo-hydroxyquinoline complexes; in vitro anti-inflammatory, antifungal, antibacterial, theoretical, and molecular docking interactions Investigation. *J. Mol. Struct* **1307**, 138016 (2024).

[18] Kasare, M. S., Dhavan, P. P., Jadhav, B. L. & Pawar, S. D. In-vitro antibacterial activity of Ni(II), Cu(II), and Zn(II) complexes incorporating new azo-azomethine ligand possessing excellent antioxidant, anti-inflammatory activity and protective effect of free radicals against plasmid DNA. *Synth. Commun.* **49**, 3311–3323 (2019).

[19] Hunger, K. *Industrial Dyes: Chemistry, Properties* (Wiley, 2003).

[20] Wang, X., Feng, L. & Chen, Z. Synthesis and photophysics of novel 8-hydroxyquinoline aluminum metal dye with hole transfer groups, *Spectrochim*. *Acta Part A Mol. Biomol. Spectrosc.* **71**, 1433–1437 (2008). 10.1016/j.saa.2008.04.026 18550423

[21] Jasim, D. J. & Abbas, A. K. Synthesis, identification, antibacterial, medical and dying performance studies for azo-sulfamethoxazole metal complexes. *Eurasian Chem. Commun.* **4**, 16–40 (2022).

[22] El-Sonbati, A. Z., Diab, M. A., Morgan, S. M., Abou-Dobara, M. I. & El-Ghettany, A. A. Synthesis, characterization, theoretical and molecular docking studies of mixed-ligand complexes of Cu(II), Ni(II), Co(II), Mn(II), Cr(III), UO2(II) and Cd(II). *J. Mol. Struct.* **1200**, 127065 (2020).

[23] Gaber, M., El-Hefnawy, G. B., El-Borai, M. A. & Mohamed, N. F. Synthesis, spectral and thermal studies of Mn(II), Co(II), Ni(II), Cu(II) and Zn(II) complex dyes based on hydroxyquinoline moiety. *J. Therm. Anal. Calorim.* **109**, 1397–1405 (2012).

[24] Maity, A., Sinha, D. & Rajak, K. K. Experimental and theoretical studies of structural and photophysical properties of a novel heteroleptic cyclometalated iridium(III) complex with 8-hydroxyquinoline-phenylazo ligand. *J. Mol. Struct.* **1158**, 122–132 (2018).

[25] Zidan, A. Studies on some transition metal mixed ligands complexes Glycinyldithiocarbamate and 8-hydroxyquinoline moiety. *J. Therm. Anal. Calorim.* **68**, 1045–1059 (2002).

[26] Ihara, T. et al. Metal ion-directed cooperative DNA binding of small molecules. *J. Inorg. Biochem.* **100**, 1744–1754 (2006). 16904185 10.1016/j.jinorgbio.2006.06.008

[27] La Deda, M., Grisolia, A., Aiello, I., Crispini, A., Ghedini, M., Belviso, S., Amati, M. & Lelj, F. Investigations on the electronic effects of the peripheral 4′-group on 5-(4′-substituted)phenylazo-8-hydroxyquinoline ligands: Zinc and aluminium complexes. *J. Chem. Soc. Dalt. Trans.* **2004**, 2424–2431. 10.1039/b404814h 15303154

[28] Shoair, A. F., El-Bindary, A. A., El-Sonbati, A. Z. & Younes, R. M. Stereochemistry of new nitrogen containing heterocyclic aldehyde. VI. Novel structural and properties models of uranyl with quinoline azodyes, Spectrochim. *Acta Part A Mol. Biomol. Spectrosc.* **57**, 1683–1691 (2001). 10.1016/s1386-1425(01)00398-5 11471721

[29] Mekheimer, R., Ahmed, E. K. & Khattab, A. F. A novel nucleophilic substitution with quinoline. Derivatives synthesis of quinolones and Pyrazolo[4,3-c]quinoline Derivatives. *Bull. Chem. Soc. Jpn.* **66**, 2936–2940 (1993).

[30] Hartline, C. B. et al. Inhibition of herpesvirus replication by a series of 4-oxo-dihydroquinolines with viral polymerase activity. *Antivir. Res.* **65**, 97–105 (2005). 15708636 10.1016/j.antiviral.2004.10.003

[31] Pelletier, C., Prognon, P. & Bourlioux, P. Roles of divalent cations and ph in mechanism of action of Nitroxoline against escherichia coli strains. *Antimicrob. Agents Chemother.* **39**, 707–713 (1995). 7793877 10.1128/AAC.39.3.707 PMC162609

[32] Sobke, A. et al. The urinary antibiotic 5-Nitro-8-hydroxyquinoline (Nitroxoline) reduces the formation and induces the dispersal of pseudomonas aeruginosa biofilms by chelation of iron and zinc. *Antimicrob. Agents Chemother.* **56**, 6021–6025 (2012). 22926564 10.1128/AAC.01484-12 PMC3486607

[33] Cancet, B. & Amgar, A. Activite´ antifongique de la nitroxoline in vitro. Re´sultats cliniques pre´liminaires. *Pathol. Biol. Paris.* **35**, 879–881 (1987). 3309833

[34] Shim, J. S. et al. Effect of Nitroxoline on angiogenesis and growth of human bladder cancer. *J. Natl. Cancer Inst.* **102**, 1855–1873 (2010). 21088277 10.1093/jnci/djq457 PMC3001967

[35] El-Wakiel, N. & El-Ghamry, H. Nitroxoline azo dye complexes as effective heterogeneous catalysts for color removal and degradation of some organic textile dyes. *Int. J. Chem. Kinet.* **49**, 464–476 (2017).

[36] El-Wakiel, N. A. Synthesis and characterization of azo sulfaguanidine complexes and their application for corrosion inhibition of silicate glass. *Appl. Organomet. Chem.* **30**, 664–673 (2016).

[37] El-Wakiel, N. A., Rizk, H. F. & Ibrahim, S. A. Synthesis and characterization of metal complexes of azo dye based on 5-nitro-8-hydroxyquinoline and their applications in dyeing polyester fabrics. *Appl. Organomet. Chem.* **31**, e3723 (2017).

[38] Gaber, M., El-Wakiel, N. & Hemeda, O. M. Cr (III), Mn (II), Co (II), Ni (II) and Cu (II) complexes of 7-((1H-benzo [d] imidazol-2-yl) diazenyl)-5-nitroquinolin-8-ol. synthesis, thermal, spectral, electrical. *J. Mol. Struct.* **1180**, 318–329 (2019).

[39] Fetter, T. et al. The role of adenosine in the pathogenesis of rheumatoid arthritis. *Front. Immunol.* **11**, 344 (2020). 32194562 10.3389/fimmu.2020.00344 PMC7064060

[40] Haskó, G. & Cronstein, B. N. Adenosine: An endogenous regulator of innate immunity. *Trends Immunol.* **25**, 33–39 (2004). 14698282 10.1016/j.it.2003.11.003

[41] Allard, B., Allard, D., Buisseret, L. & Stagg, J. The adenosine pathway in immuno-oncology. *Nat. Rev. Clin. Oncol.* **17**, 611–629 (2020). 32514148 10.1038/s41571-020-0382-2

[42] Antonioli, L. et al. Inflammatory bowel diseases: It’s time for the adenosine system. *Front. Immunol.* **11**, 1310 (2020). 32849492 10.3389/fimmu.2020.01310 PMC7403190

[43] El-Said, K. S. et al. Quercetin mitigates rheumatoid arthritis by inhibiting adenosine deaminase in rats. *Mol. Med.* **28**, 24 (2022). 35193490 10.1186/s10020-022-00432-5 PMC8862293

[44] Cronstein, B. N. & Sitkovsky, M. Adenosine and adenosine receptors in the pathogenesis and treatment of rheumatic diseases. *Nat. Rev. Rheumatol.* **13**, 41–51 (2017). 27829671 10.1038/nrrheum.2016.178 PMC5173391

[45] Jacobson, K. A. & Gao, Z.-G. Adenosine receptors as therapeutic targets. *Nat. Rev. Drug Discov.* **5**, 247–264 (2006). 16518376 10.1038/nrd1983 PMC3463109

[46] Deaglio, S. & Robson, S. C. Chapter 10—Ectonucleotidases as regulators of purinergic signaling in thrombosis, inflammation, and immunity. *Adv. Pharmacol* **61**, 301–332 (2011). 21586363 10.1016/B978-0-12-385526-8.00010-2 PMC5879773

[47] Kutryb-Zajac, B., Mierzejewska, P., Slominska, E. M. & Smolenski, R. T. Therapeutic perspectives of adenosine deaminase inhibition in cardiovascular diseases. *Molecules* **25**, 4652 (2020). 33053898 10.3390/molecules25204652 PMC7587364

[48] Mohamed, T. M. Adenosine deaminase from camel tick Hyalomma dromedarii: Purification and characterization. *Exp. Appl. Acarol.* **40**, 101–111 (2006). 17089216 10.1007/s10493-006-9023-4

[49] Babaker, M. M., Khalid, M. & Al-mukhtar, S. E. Synthesis, Characterization, and computational study of novel 2-Phenoxyethyl xanthate ligand and complexes with some transition metals. *Orient. J. Chem.* **2023**, 39.

[50] Fetoh, A., Salah, Z. & Abu El-Reash, G. M. Structural studies and biological evaluation of Co (II), Ni (II) and Cu (II) complexes of carbohydrazone derived from ethyl acetoacetate in addition to crystallographic description of La (III) or Sm (III) catalytic activity abnormal product. *Appl. Organomet. Chem* **33**, e4727 (2019).

[51] Molecular operating environment (MOE) 2014.09, Chemical Computing Group Inc., 1010 Sherbrooke Street West, Suite 910, Montréal, H3A 2R7, Canada, http://www.chemcomp.com/

[52] Bradford, M. M. A rapid and sensitive method for the quantitation of microgram quantities of protein utilizing the principle of protein-dye binding. *Anal. Biochem.* **72**, 248–254 (1976). 942051 10.1016/0003-2697(76)90527-3

[53] Abu-Khudir, R., Salem, M. M., Allam, N. G. & Ali, E. M. M. Production, partial purification, and biochemical characterization of a thermotolerant alkaline metallo-protease from Staphylococcus sciuri. *Appl. Biochem. Biotechnol.* **189**, 87–102 (2019). 30868382 10.1007/s12010-019-02983-6

[54] Alhasani, M. A., Farghaly, T. A. & El-Ghamry, H. A. Mono- and bimetallic complexes of pyrazolone based ligand: Synthesis, characterization, antitumor and molecular docking studies. *J. Mol. Struct.* **1249**, 131607 (2022).

[55] El-Ghamry, H., El-Wakiel, N. & Khamis, A. Synthesis, structure, antiproliferative activity and molecular docking of divalent and trivalent metal complexes of 4*H*–3,5-diamino-1,2,4-triazole and α-hydroxynaphthaldehyde Schiff base ligand. *Appl. Organomet. Chem.* **32**, e4583 (2018).

[56] Kanaani, A., Ajloo, D., Grivani, G., Ghavami, A. & Vakili, M. Tautomeric stability, Tautomeric stability, molecular structure, NBO, electronic and NMR analyses of salicylideneimino-ethylimino-pentan-2- one. *J. Mol. Struct* **1112**, 87–96 (2016).

[57] De Lorenzi, A., Giorgianni, S. & Bini, R. High-resolution FTIR spectroscopy of the C—Cl stretching mode of vinyl chloride. *Mol. Phys.* **96**, 101–108 (1999).

[58] Amer, S., El-Wakiel, N. & El-Ghamry, H. Synthesis, spectral, antitumor and antimicrobial studies on Cu(II) complexes of purine and triazole Schiff base derivatives. *J. Mol. Struct.* **1049**, 326–335 (2013).

[59] Song, Y. et al. Solution studies and structure of a dinuclear-based double-stranded helicate of [Cu2(p-xysal)2] (H2-p-xysal=bis(hydroxylbenzyl)diaminoxylene). *Polyhedron* **23**, 1769–1775 (2004).

[60] Shimizu, I. et al. Tetrahedral copper(II) complexes with a labile coordination site supported by a Tris-tetramethylguanidinato ligand. *Inorg. Chem.* **56**, 9634–9645 (2017). 28753281 10.1021/acs.inorgchem.7b01154

[61] Saad, F. A. et al. Elaborated spectral, modeling, QSAR, docking, thermal, antimicrobial and anticancer activity studies for new nanosized metal ion complexes derived from sulfamerazine azodye. *J. Therm. Anal. Calorim.* **131**, 1249–1267 (2018).

[62] El-wakiel, N., El-keiy, M. & Gaber, M. Synthesis, spectral, antitumor, antioxidant and antimicrobial studies on Cu(II), Ni(II) and Co(II) complexes of 4-[(1H-Benzoimidazol-2-ylimino)- methyl]-benzene-1,3-diol. *Spectrochim. Acta Part A Mol. Biomol. Spectrosc.* **147**, 117–123 (2015). 10.1016/j.saa.2015.03.020 25827773

[63] Yarkandi, N. H., El-Ghamry, H. A. & Gaber, M. Synthesis, spectroscopic and DNA binding ability of CoII, NiII, CuII and ZnII complexes of Schiff base ligand (E)-1-(((1H-benzo[d]imidazol-2-yl)methylimino)methyl)naphthalen-2-ol. X-ray crystal structure determination of cobalt(II) complex. *Mater. Sci. Eng. C* **75**, 1059–1067 (2017). 10.1016/j.msec.2017.02.171 28415390

[64] El-Sherif, A. A., Fetoh, A., Abdulhamed, Y. K. & El-Reash, G. M. A. Synthesis, structural characterization, DFT studies and biological activity of Cu(II) and Ni(II) complexes of novel hydrazone. *Inorganica Chim. Acta* **480**, 1–15 (2018).

[65] Allen, F. H. & Bruno, I. J. Bond lengths in organic and metal-organic compounds revisited: X—H bond lengths from neutron diffraction data. *Acta Crystallogr. Sect. B Struct. Sci.* **66**, 380–386 (2010). 10.1107/S0108768110012048 20484809

[66] El-Reash, G. M. A., El-Gammal, O. A., Ghazy, S. E. & Radwan, A. H. Characterization and biological studies on Co (II), Ni (II) and Cu (II) complexes of carbohydrazones ending by pyridyl ring, *Spectrochim*. *Acta Part A Mol. Biomol. Spectrosc.* **104**, 26–34 (2013). 10.1016/j.saa.2012.11.008 23274253

[67] Gaber, M., Fayed, T. A., El-Gamil, M. M. & El-Reash, G. M. A. Structural, thermogravimetric, B3LYP and biological studies on some heterocyclic thiosemicarbazide copper (II) complexes and evaluation of their molecular docking. *J. Mol. Struct.* **1151**, 56–72 (2018).

[68] Fouda, A.E.-A.S., El-Askalany, A. H., Molouk, A. F. S., Elsheikh, N. S. & Abousalem, A. S. Experimental and computational chemical studies on the corrosion inhibitive properties of carbonitrile compounds for carbon steel in aqueous solutions. *Sci. Rep.* **11**, 21672 (2021). 34737347 10.1038/s41598-021-00701-z PMC8569179

[69] Gong, Y., Wang, Z., Gao, F., Zhang, S. & Li, H. Synthesis of new benzotriazole derivatives containing carbon chains as the corrosion inhibitors for copper in sodium chloride solution. *Ind. Eng. Chem. Res.* **54**, 12242–12253 (2015).

[70] Guo, L. et al. Toward understanding the adsorption mechanism of large size organic corrosion inhibitors on an Fe(110) surface using the DFTB method. *RSC Adv.* **7**, 29042–29050 (2017).

