# Supplementary material for: Synthesis, characterization and theoretical studies of nitroxoline azo dye metal complexes and their role in mitigation of rheumatoid arthritis
Source: Sci Rep. 2025 Jun 20;15:20213. doi: 10.1038/s41598-025-06518-4 (PMC12181347; doi:10.1038/s41598-025-06518-4)
Supplement: Supplementary file 1 — Supplementary Material 1 [file 41598_2025_6518_MOESM1_ESM.docx]

**Synthesis, characterization, theoretical and molecular docking studies of nitroxoline-based metal complexes and their role in mitigation of rheumatoid arthritis**

**Hoda A. El-Ghamry^1*^ | Mohamed Gaber^1^ |** **Mariam S. Anis^1^ | Amira Atta^2^ | Tarek M. Mohamed^2^ | Nadia A. El‐Wakiel^1^**

^1^Chemistry Department, Faculty of Science, Tanta University, Tanta 31527, Egypt

^2^Biochemistry Division, Chemistry Department, Faculty of Science, Tanta University, Tanta 31527, Egypt

* corresponding author e-mail address: [hoda.elghamri@science.tanta.edu.eg](mailto:hoda.elghamri@science.tanta.edu.eg)

**Section S1: Instruments and methods**

Elemental microanalysis for C, H and N percentages were achieved by a 2400 CHN Elemental Analyzer (Perkin-Elmer). The ^1^HNMR spectra were carried out using a Varian Mercury Oxford NMR 300 Hz spectrophotometer after dissolving the samples in d^6^-DMSO using tetramethylsilane (TMS) as internal standard . Standard electron impact mass spectra (E.I) of the ligands and some of their metal complexes were determined using a Finnigan MAT 8222 Spectrometer at 70 eV at Micro Analytical. Sherwood magnetic susceptibility balance was applied to attain the room temperature magnetic moment values of the complexes. Electronic absorption spectra of all compounds were performed by applying Nujol mull using T80+Uv/Vis spectrometer within the range 200-800 nm.. The molar conductance was determined in a DMF solvent (10^-3^ mol L^-1^) using a JENWAY (model 4070) conductance bridge. FT-IR Bruker Tensor 27 spectrophotometer was used to measure FT-IR spectra as KBr disks within a 4000–200 cm^−1^ range. The thermo-gravimetric analysis (TGA) of complexes was accomplished using a Shimadzu TG-50 thermal analyzer, in the presence of N_2_ as atmosphere using 10 ^ο^C/min heating rate.

**Table S1.** List of bond angles for the optimized structure of ligand **CPAQ**

| Atom1 | Atom2 | Atom3 | Angle | Atom1 | Atom2 | Atom3 | Angle |
| --- | --- | --- | --- | --- | --- | --- | --- |
| C2 | C1 | C6 | 117.88 | C16 | C17 | C18 | 120.24 |
| C2 | C1 | C7 | 118.58 | C16 | C17 | H30 | 119.83 |
| C6 | C1 | C7 | 123.54 | C18 | C17 | H30 | 119.93 |
| C1 | C2 | N3 | 122.99 | C17 | C18 | C19 | 120.31 |
| C1 | C2 | C10 | 120.31 | C17 | C18 | H31 | 119.8 |
| N3 | C2 | C10 | 116.7 | C19 | C18 | H31 | 119.88 |
| C2 | N3 | C4 | 118.01 | C18 | C19 | C20 | 119.87 |
| N3 | C4 | C5 | 121.76 | C18 | C19 | H32 | 120.36 |
| N3 | C4 | H25 | 118.19 | C20 | C19 | H32 | 119.78 |
| C5 | C4 | H25 | 120.05 | C19 | C20 | C15 | 120.87 |
| C4 | C5 | C6 | 120.12 | C19 | C20 | Cl22 | 119.56 |
| C4 | C5 | H26 | 119.93 | C15 | C20 | Cl22 | 119.57 |
| C6 | C5 | H26 | 119.96 | C7 | N12 | O23 | 122.59 |
| C5 | C6 | C1 | 119.24 | C7 | N12 | O24 | 118.27 |
| C5 | C6 | H27 | 120.53 | O23 | N12 | O24 | 119.14 |
| C1 | C6 | H27 | 120.24 | C9 | N13 | N14 | 115.95 |
| C1 | C7 | C8 | 120.41 | N13 | N14 | C15 | 117.57 |
| C1 | C7 | N12 | 124.27 | N14 | C15 | C16 | 125.95 |
| C8 | C7 | N12 | 115.32 | N14 | C15 | C20 | 115.33 |
| C7 | C8 | C9 | 120.68 | C16 | C15 | C20 | 118.71 |
| C7 | C8 | H28 | 119.87 | C15 | C16 | C17 | 120 |
| C9 | C8 | H28 | 119.44 | C15 | C16 | Cl21 | 122.67 |
| C8 | C9 | C10 | 119.47 | C17 | C16 | Cl21 | 117.33 |
| C8 | C9 | N13 | 116.62 | C9 | C10 | O11 | 120.45 |
| C10 | C9 | N13 | 123.89 | C2 | C10 | O11 | 119.02 |
| C9 | C10 | C2 | 120.52 | C10 | O11 | H29 | 106.3 |

**Table S2.** List of bond lengths for the optimized structure of ligand **CPAQ**

| Atom1 | Atom2 | Type | Length |
| --- | --- | --- | --- |
| C1 | C2 | Single | 1.4978 |
| C2 | N3 | Double | 1.4559 |
| N3 | C4 | Single | 1.4437 |
| C4 | C5 | Double | 1.4866 |
| C5 | C6 | Single | 1.4768 |
| C1 | C6 | Double | 1.4882 |
| C1 | C7 | Single | 1.5102 |
| C7 | C8 | Double | 1.4827 |
| C8 | C9 | Single | 1.4815 |
| C9 | C10 | Double | 1.4917 |
| C2 | C10 | Single | 1.4902 |
| C10 | O11 | Single | 1.4513 |
| C7 | N12 | Single | 1.4739 |
| C9 | N13 | Single | 1.4658 |
| N13 | N14 | Double | 1.4117 |
| N14 | C15 | Single | 1.4702 |
| C15 | C16 | Double | 1.5074 |
| C16 | C17 | Single | 1.4832 |
| C17 | C18 | Double | 1.4814 |
| C18 | C19 | Single | 1.481 |
| C19 | C20 | Double | 1.4802 |
| C20 | C15 | Single | 1.4918 |
| C16 | Cl21 | Single | 1.7463 |
| C20 | Cl22 | Single | 1.7466 |
| N12 | O23 | Double | 1.4129 |
| N12 | O24 | Double | 1.4139 |
| C4 | H25 | Single | 1.1209 |
| C5 | H26 | Single | 1.1214 |
| C6 | H27 | Single | 1.1189 |
| C8 | H28 | Single | 1.1207 |
| O11 | H29 | Single | 1.0681 |
| C17 | H30 | Single | 1.1211 |
| C18 | H31 | Single | 1.121 |
| C19 | H32 | Single | 1.121 |

**Table S3.** List of bond angles for the optimized structure of ligand **CPAQ-Co**

| Atom1 | Atom2 | Atom3 | Angle | Atom1 | Atom2 | Atom3 | Angle |
| --- | --- | --- | --- | --- | --- | --- | --- |
| C2 | N1 | N22 | 113.71 | C13 | C14 | C19 | 116.25 |
| C2 | N1 | Co23 | 118.47 | C15 | C14 | C19 | 117.34 |
| N22 | N1 | Co23 | 127.18 | C14 | C15 | C16 | 121.1 |
| N1 | C2 | C3 | 120.03 | C14 | C15 | N21 | 123.35 |
| N1 | C2 | C7 | 121.57 | C16 | C15 | N21 | 115.55 |
| C3 | C2 | C7 | 118.26 | C15 | C16 | C17 | 122.98 |
| C2 | C3 | C4 | 121.42 | C15 | C16 | H36 | 119.7 |
| C2 | C3 | Cl9 | 119.21 | C17 | C16 | H36 | 117.31 |
| C4 | C3 | Cl9 | 119.36 | C16 | C17 | C18 | 119.21 |
| C3 | C4 | C5 | 119.13 | C16 | C17 | N22 | 112.59 |
| C3 | C4 | H30 | 119.88 | C18 | C17 | N22 | 128.19 |
| C5 | C4 | H30 | 120.99 | C17 | C18 | C19 | 117.68 |
| C4 | C5 | C6 | 120.76 | C17 | C18 | O20 | 124.15 |
| C4 | C5 | H31 | 119.71 | C19 | C18 | O20 | 118.17 |
| C6 | C5 | H31 | 119.53 | C18 | C19 | N10 | 115.03 |
| C5 | C6 | C7 | 119.61 | C18 | C19 | C14 | 121.64 |
| C5 | C6 | H32 | 120.91 | N10 | C19 | C14 | 123.33 |
| C7 | C6 | H32 | 119.47 | C18 | O20 | Co23 | 124.06 |
| C6 | C7 | C2 | 120.81 | C15 | N21 | O28 | 118.28 |
| C6 | C7 | Cl8 | 119.06 | C15 | N21 | O29 | 118.96 |
| C2 | C7 | Cl8 | 120.11 | O28 | N21 | O29 | 122.74 |
| C11 | N10 | C19 | 118.45 | N1 | N22 | C17 | 121.3 |
| N10 | C11 | C12 | 122.88 | O20 | Co23 | Cl24 | 120.04 |
| N10 | C11 | H33 | 116.56 | O20 | Co23 | N1 | 94.72 |
| C12 | C11 | H33 | 120.56 | O20 | Co23 | O25 | 101.78 |
| C11 | C12 | C13 | 119.3 | Cl24 | Co23 | N1 | 116.81 |
| C11 | C12 | H34 | 120.22 | Cl24 | Co23 | O25 | 105.89 |
| C13 | C12 | H34 | 120.48 | N1 | Co23 | O25 | 117.02 |
| C12 | C13 | C14 | 119.74 | H26 | O25 | H27 | 105.11 |
| C12 | C13 | H35 | 120.08 | H26 | O25 | Co23 | 115.65 |
| C14 | C13 | H35 | 120.17 | H27 | O25 | Co23 | 114.83 |
| C13 | C14 | C15 | 126.38 |  |  |  |  |

**Table S4.** List of bond lengths for the optimized structure of ligand **CPAQ-Co**

| Atom1 | Atom2 | Type | Length | Atom1 | Atom2 | Type | Length |
| --- | --- | --- | --- | --- | --- | --- | --- |
| N1 | C2 | Single | 1.4346 | C18 | O20 | Single | 1.2847 |
| C2 | C3 | Single | 1.4064 | C15 | N21 | Single | 1.4627 |
| C3 | C4 | Double | 1.3915 | N1 | N22 | Double | 1.288 |
| C4 | C5 | Single | 1.3959 | N22 | C17 | Single | 1.3703 |
| C5 | C6 | Double | 1.3955 | O20 | Co23 | Single | 1.9213 |
| C6 | C7 | Single | 1.3944 | Co23 | Cl24 | Single | 2.2018 |
| C2 | C7 | Double | 1.4068 | N1 | Co23 | Single | 1.9349 |
| C7 | Cl8 | Single | 1.7414 | O25 | H26 | Single | 0.9766 |
| C3 | Cl9 | Single | 1.746 | O25 | H27 | Single | 0.9767 |
| N10 | C11 | Double | 1.329 | O25 | Co23 | Single | 2.0553 |
| C11 | C12 | Single | 1.4054 | N21 | O28 | Double | 1.2442 |
| C12 | C13 | Double | 1.3835 | N21 | O29 | Double | 1.2445 |
| C13 | C14 | Single | 1.4155 | C4 | H30 | Single | 1.0886 |
| C14 | C15 | Single | 1.4471 | C5 | H31 | Single | 1.09 |
| C15 | C16 | Double | 1.371 | C6 | H32 | Single | 1.0886 |
| C16 | C17 | Single | 1.4143 | C11 | H33 | Single | 1.093 |
| C17 | C18 | Double | 1.4424 | C12 | H34 | Single | 1.0896 |
| C18 | C19 | Single | 1.4671 | C13 | H35 | Single | 1.0842 |
| N10 | C19 | Single | 1.3533 | C16 | H36 | Single | 1.0869 |
| C14 | C19 | Double | 1.432 |  |  |  |  |

**Table S5.** List of bond angles for the optimized structure of ligand **CPAQ-Ni**

| Atom1 | Atom2 | Atom3 | Angle | Atom1 | Atom2 | Atom3 | Angle |
| --- | --- | --- | --- | --- | --- | --- | --- |
| C2 | N1 | N22 | 111.49 | C17 | C16 | H39 | 119.77 |
| C2 | N1 | Ni23 | 129.8 | C16 | C17 | C18 | 119.59 |
| N22 | N1 | Ni23 | 117.11 | C16 | C17 | N22 | 116.39 |
| N1 | C2 | C3 | 126.12 | C18 | C17 | N22 | 123.99 |
| N1 | C2 | C7 | 118.38 | C17 | C18 | C19 | 120.25 |
| C3 | C2 | C7 | 115.49 | C17 | C18 | O20 | 120.44 |
| C2 | C3 | C4 | 120.52 | C19 | C18 | O20 | 119.26 |
| C2 | C3 | Cl9 | 123.92 | C18 | C19 | N10 | 117.3 |
| C4 | C3 | Cl9 | 115.01 | C18 | C19 | C14 | 120.3 |
| C3 | C4 | C5 | 120.72 | N10 | C19 | C14 | 122.39 |
| C3 | C4 | H33 | 119.34 | C18 | O20 | Ni23 | 109.14 |
| C5 | C4 | H33 | 119.87 | C15 | N21 | O27 | 118.56 |
| C4 | C5 | C6 | 119.43 | C15 | N21 | O28 | 122.3 |
| C4 | C5 | H34 | 120.27 | O27 | N21 | O28 | 119.14 |
| C6 | C5 | H34 | 120.24 | N1 | N22 | C17 | 122.97 |
| C5 | C6 | C7 | 120.25 | O20 | Ni23 | Cl24 | 178.37 |
| C5 | C6 | H35 | 120.07 | O20 | Ni23 | N1 | 90.18 |
| C7 | C6 | H35 | 119.67 | O20 | Ni23 | O25 | 90.13 |
| C6 | C7 | C2 | 121.23 | O20 | Ni23 | O31 | 90.9 |
| C6 | C7 | Cl8 | 115.67 | O20 | Ni23 | O29 | 87.91 |
| C2 | C7 | Cl8 | 123.08 | Cl24 | Ni23 | N1 | 91.42 |
| C11 | N10 | C19 | 118.41 | Cl24 | Ni23 | O25 | 88.27 |
| N10 | C11 | C12 | 121.72 | Cl24 | Ni23 | O31 | 88.78 |
| N10 | C11 | H36 | 118.27 | Cl24 | Ni23 | O29 | 92.35 |
| C12 | C11 | H36 | 120.01 | N1 | Ni23 | O25 | 179.4 |
| C11 | C12 | C13 | 119.93 | N1 | Ni23 | O31 | 89.27 |
| C11 | C12 | H37 | 120.13 | N1 | Ni23 | O29 | 93.14 |
| C13 | C12 | H37 | 119.95 | O25 | Ni23 | O31 | 90.21 |
| C12 | C13 | C14 | 119.32 | O25 | Ni23 | O29 | 87.39 |
| C12 | C13 | H38 | 120.37 | O31 | Ni23 | O29 | 177.31 |
| C14 | C13 | H38 | 120.31 | H26 | O25 | Ni23 | 109.68 |
| C13 | C14 | C15 | 122.96 | H26 | O25 | H40 | 108.73 |
| C13 | C14 | C19 | 118.24 | Ni23 | O25 | H40 | 110.14 |
| C15 | C14 | C19 | 118.8 | H30 | O29 | Ni23 | 109.7 |
| C14 | C15 | C16 | 120.31 | H30 | O29 | H41 | 108.6 |
| C14 | C15 | N21 | 123.88 | Ni23 | O29 | H41 | 109.4 |
| C16 | C15 | N21 | 115.82 | H32 | O31 | Ni23 | 109.88 |
| C15 | C16 | C17 | 120.71 | H32 | O31 | H42 | 108.1 |
| C15 | C16 | H39 | 119.52 | Ni23 | O31 | H42 | 109.99 |

**Table S6.** List of bond lengths for the optimized structure of ligand **CPAQ-Ni**

| Atom1 | Atom2 | Type | Length | Atom1 | Atom2 | Type | Length |
| --- | --- | --- | --- | --- | --- | --- | --- |
| N1 | C2 | Single | 1.51 | N22 | C17 | Single | 1.4765 |
| C2 | C3 | Single | 1.5192 | O20 | Ni23 | Single | 2.0979 |
| C3 | C4 | Double | 1.4885 | Ni23 | Cl24 | Single | 2.3582 |
| C4 | C5 | Single | 1.4803 | N1 | Ni23 | Single | 2.1521 |
| C5 | C6 | Double | 1.4799 | O25 | H26 | Single | 1.0675 |
| C6 | C7 | Single | 1.4883 | O25 | Ni23 | Single | 2.1159 |
| C2 | C7 | Double | 1.5223 | N21 | O27 | Double | 1.4215 |
| C7 | Cl8 | Single | 1.7498 | N21 | O28 | Double | 1.4201 |
| C3 | Cl9 | Single | 1.7445 | O29 | H30 | Single | 1.067 |
| N10 | C11 | Double | 1.451 | O31 | H32 | Single | 1.0669 |
| C11 | C12 | Single | 1.4906 | O31 | Ni23 | Single | 2.1213 |
| C12 | C13 | Double | 1.4822 | O29 | Ni23 | Single | 2.1092 |
| C13 | C14 | Single | 1.4934 | C4 | H33 | Single | 1.1229 |
| C14 | C15 | Single | 1.5144 | C5 | H34 | Single | 1.1228 |
| C15 | C16 | Double | 1.4888 | C6 | H35 | Single | 1.1226 |
| C16 | C17 | Single | 1.4885 | C11 | H36 | Single | 1.1229 |
| C17 | C18 | Double | 1.4986 | C12 | H37 | Single | 1.1237 |
| C18 | C19 | Single | 1.4999 | C13 | H38 | Single | 1.1185 |
| N10 | C19 | Single | 1.462 | C16 | H39 | Single | 1.1226 |
| C14 | C19 | Double | 1.5024 | O25 | H40 | Single | 1.0674 |
| C18 | O20 | Single | 1.4369 | O29 | H41 | Single | 1.0678 |
| C15 | N21 | Single | 1.4767 | O31 | H42 | Single | 1.0675 |
| N1 | N22 | Double | 1.4356 |  |  |  |  |

**Table S7.** List of bond angles for the optimized structure of ligand **CPAQ-Cu**

| Atom1 | Atom2 | Atom3 | Angle | Atom1 | Atom2 | Atom3 | Angle | Atom1 | Atom2 | Atom3 | Angle |
| --- | --- | --- | --- | --- | --- | --- | --- | --- | --- | --- | --- |
| C2 | N1 | N22 | 114.15 | C17 | C16 | H56 | 119.66 | C29 | C30 | C25 | 120.24 |
| C2 | N1 | Cu23 | 123.97 | C16 | C17 | C18 | 119.42 | C29 | C30 | Cl31 | 118.21 |
| N22 | N1 | Cu23 | 119.47 | C16 | C17 | N22 | 115.63 | C25 | C30 | Cl31 | 121.43 |
| N1 | C2 | C3 | 121.19 | C18 | C17 | N22 | 124.92 | C34 | N33 | C42 | 118.99 |
| N1 | C2 | C7 | 119.82 | C17 | C18 | C19 | 119.85 | N33 | C34 | C35 | 121.35 |
| C3 | C2 | C7 | 118.98 | C17 | C18 | O20 | 122.17 | N33 | C34 | H60 | 118.68 |
| C2 | C3 | C4 | 120.13 | C19 | C18 | O20 | 117.85 | C35 | C34 | H60 | 119.96 |
| C2 | C3 | Cl9 | 120.72 | C18 | C19 | N10 | 117.89 | C34 | C35 | C36 | 119.88 |
| C4 | C3 | Cl9 | 118.96 | C18 | C19 | C14 | 120.34 | C34 | C35 | H61 | 120.01 |
| C3 | C4 | C5 | 120.06 | N10 | C19 | C14 | 121.74 | C36 | C35 | H61 | 120.1 |
| C3 | C4 | H50 | 119.79 | C18 | O20 | Cu23 | 115.04 | C35 | C36 | C37 | 119.11 |
| C5 | C4 | H50 | 120.15 | C15 | N21 | O46 | 118.8 | C35 | C36 | H62 | 120.44 |
| C4 | C5 | C6 | 120.31 | C15 | N21 | O47 | 121.91 | C37 | C36 | H62 | 120.45 |
| C4 | C5 | H51 | 119.86 | O46 | N21 | O47 | 119.27 | C36 | C37 | C38 | 122.28 |
| C6 | C5 | H51 | 119.82 | N1 | N22 | C17 | 123.51 | C36 | C37 | C42 | 118.76 |
| C5 | C6 | C7 | 119.81 | O20 | Cu23 | O43 | 106.09 | C38 | C37 | C42 | 118.95 |
| C5 | C6 | H52 | 120.27 | O20 | Cu23 | N1 | 98.61 | C37 | C38 | C39 | 120.06 |
| C7 | C6 | H52 | 119.92 | O20 | Cu23 | N24 | 113.44 | C37 | C38 | N44 | 122.8 |
| C6 | C7 | C2 | 120.33 | O43 | Cu23 | N1 | 111.53 | C39 | C38 | N44 | 117.13 |
| C6 | C7 | Cl8 | 118.62 | O43 | Cu23 | N24 | 99.06 | C38 | C39 | C40 | 120.59 |
| C2 | C7 | Cl8 | 120.97 | N1 | Cu23 | N24 | 127.04 | C38 | C39 | H63 | 119.64 |
| C11 | N10 | C19 | 119.18 | C25 | N24 | N45 | 115.67 | C40 | C39 | H63 | 119.77 |
| N10 | C11 | C12 | 121.29 | C25 | N24 | Cu23 | 122.78 | C39 | C40 | C41 | 119.03 |
| N10 | C11 | H53 | 118.76 | N45 | N24 | Cu23 | 119.75 | C39 | C40 | N45 | 116.43 |
| C12 | C11 | H53 | 119.95 | N24 | C25 | C26 | 121.09 | C41 | C40 | N45 | 124.53 |
| C11 | C12 | C13 | 119.62 | N24 | C25 | C30 | 120.51 | C40 | C41 | C42 | 119.53 |
| C11 | C12 | H54 | 120.27 | C26 | C25 | C30 | 118.4 | C40 | C41 | O43 | 121.15 |
| C13 | C12 | H54 | 120.11 | C25 | C26 | C27 | 120.43 | C42 | C41 | O43 | 119.25 |
| C12 | C13 | C14 | 119.58 | C25 | C26 | Cl32 | 120.75 | C41 | C42 | N33 | 117.98 |
| C12 | C13 | H55 | 120.21 | C27 | C26 | Cl32 | 118.42 | C41 | C42 | C37 | 120.16 |
| C14 | C13 | H55 | 120.22 | C26 | C27 | C28 | 120.22 | N33 | C42 | C37 | 121.86 |
| C13 | C14 | C15 | 122.22 | C26 | C27 | H57 | 119.73 | C41 | O43 | Cu23 | 111.41 |
| C13 | C14 | C19 | 118.59 | C28 | C27 | H57 | 120.01 | C38 | N44 | O48 | 121.51 |
| C15 | C14 | C19 | 119.19 | C27 | C28 | C29 | 119.82 | C38 | N44 | O49 | 118.97 |
| C14 | C15 | C16 | 120.19 | C27 | C28 | H58 | 120.07 | O48 | N44 | O49 | 119.5 |
| C14 | C15 | N21 | 123.11 | C29 | C28 | H58 | 120.09 | N24 | N45 | C40 | 122.82 |
| C16 | C15 | N21 | 116.7 | C28 | C29 | C30 | 120.18 |  |  |  |  |
| C15 | C16 | C17 | 120.73 | C28 | C29 | H59 | 120.03 |  |  |  |  |
| C15 | C16 | H56 | 119.61 | C30 | C29 | H59 | 119.78 |  |  |  |  |

**Table S8.** List of bond lengths for the optimized structure of ligand **CPAQ-Cu**

| Atom1 | Atom2 | Type | Length | Atom1 | Atom2 | Type | Length |
| --- | --- | --- | --- | --- | --- | --- | --- |
| N1 | C2 | Single | 1.4868 | C34 | C35 | Single | 1.5032 |
| C2 | C3 | Single | 1.5071 | C35 | C36 | Double | 1.4953 |
| C3 | C4 | Double | 1.4954 | C36 | C37 | Single | 1.5029 |
| C4 | C5 | Single | 1.4966 | C37 | C38 | Single | 1.5172 |
| C5 | C6 | Double | 1.4953 | C38 | C39 | Double | 1.4977 |
| C6 | C7 | Single | 1.4962 | C39 | C40 | Single | 1.5012 |
| C2 | C7 | Double | 1.5118 | C40 | C41 | Double | 1.5173 |
| C7 | Cl8 | Single | 1.748 | C41 | C42 | Single | 1.5083 |
| C3 | Cl9 | Single | 1.7478 | N33 | C42 | Single | 1.4691 |
| N10 | C11 | Double | 1.4661 | C37 | C42 | Double | 1.5101 |
| C11 | C12 | Single | 1.5037 | C41 | O43 | Single | 1.456 |
| C12 | C13 | Double | 1.4968 | C38 | N44 | Single | 1.4843 |
| C13 | C14 | Single | 1.5048 | N24 | N45 | Double | 1.4415 |
| C14 | C15 | Single | 1.5215 | N45 | C40 | Single | 1.4822 |
| C15 | C16 | Double | 1.4977 | Cu23 | O43 | Single | 1.9506 |
| C16 | C17 | Single | 1.5004 | N1 | Cu23 | Single | 1.9628 |
| C17 | C18 | Double | 1.5219 | N24 | Cu23 | Single | 1.9598 |
| C18 | C19 | Single | 1.5085 | N21 | O46 | Double | 1.4311 |
| N10 | C19 | Single | 1.474 | N21 | O47 | Double | 1.4318 |
| C14 | C19 | Double | 1.509 | N44 | O48 | Double | 1.4313 |
| C18 | O20 | Single | 1.4554 | N44 | O49 | Double | 1.4327 |
| C15 | N21 | Single | 1.4866 | C4 | H50 | Single | 1.1262 |
| N1 | N22 | Double | 1.4412 | C5 | H51 | Single | 1.1257 |
| N22 | C17 | Single | 1.4821 | C6 | H52 | Single | 1.1264 |
| O20 | Cu23 | Single | 1.9441 | C11 | H53 | Single | 1.1267 |
| N24 | C25 | Single | 1.4908 | C12 | H54 | Single | 1.1278 |
| C25 | C26 | Single | 1.5074 | C13 | H55 | Single | 1.1236 |
| C26 | C27 | Double | 1.4993 | C16 | H56 | Single | 1.1269 |
| C27 | C28 | Single | 1.4974 | C27 | H57 | Single | 1.1266 |
| C28 | C29 | Double | 1.4957 | C28 | H58 | Single | 1.1268 |
| C29 | C30 | Single | 1.5019 | C29 | H59 | Single | 1.1264 |
| C25 | C30 | Double | 1.5159 | C34 | H60 | Single | 1.127 |
| C30 | Cl31 | Single | 1.752 | C35 | H61 | Single | 1.1274 |
| C26 | Cl32 | Single | 1.7491 | C36 | H62 | Single | 1.1245 |
| N33 | C34 | Double | 1.4627 | C39 | H63 | Single | 1.1264 |

**Table S9.** List of bond angles for the optimized structure of ligand **CPAQ-Zn**

| Atom1 | Atom2 | Atom3 | Angle | Atom1 | Atom2 | Atom3 | Angle | Atom1 | Atom2 | Atom3 | Angle |
| --- | --- | --- | --- | --- | --- | --- | --- | --- | --- | --- | --- |
| C2 | N1 | N22 | 117.64 | C16 | C17 | C18 | 119.16 | C25 | C30 | Cl31 | 120.84 |
| C2 | N1 | Zn23 | 120.8 | C16 | C17 | N22 | 115.74 | C34 | N33 | C42 | 119.16 |
| N22 | N1 | Zn23 | 119.8 | C18 | C17 | N22 | 125.09 | N33 | C34 | C35 | 121.13 |
| N1 | C2 | C3 | 120.28 | C17 | C18 | C19 | 119.96 | N33 | C34 | H60 | 118.91 |
| N1 | C2 | C7 | 120.59 | C17 | C18 | O20 | 122.24 | C35 | C34 | H60 | 119.95 |
| C3 | C2 | C7 | 119.11 | C19 | C18 | O20 | 117.67 | C34 | C35 | C36 | 119.94 |
| C2 | C3 | C4 | 120.22 | C18 | C19 | N10 | 117.97 | C34 | C35 | H61 | 120.09 |
| C2 | C3 | Cl9 | 120.15 | C18 | C19 | C14 | 120.46 | C36 | C35 | H61 | 119.97 |
| C4 | C3 | Cl9 | 119.41 | N10 | C19 | C14 | 121.55 | C35 | C36 | C37 | 119.24 |
| C3 | C4 | C5 | 119.75 | C18 | O20 | Zn23 | 112.23 | C35 | C36 | H62 | 120.35 |
| C3 | C4 | H50 | 119.98 | C15 | N21 | O46 | 118.93 | C37 | C36 | H62 | 120.4 |
| C5 | C4 | H50 | 120.25 | C15 | N21 | O47 | 121.56 | C36 | C37 | C38 | 122.09 |
| C4 | C5 | C6 | 120.15 | O46 | N21 | O47 | 119.5 | C36 | C37 | C42 | 118.71 |
| C4 | C5 | H51 | 119.9 | N1 | N22 | C17 | 121.75 | C38 | C37 | C42 | 119.2 |
| C6 | C5 | H51 | 119.93 | O20 | Zn23 | O43 | 106.91 | C37 | C38 | C39 | 120.27 |
| C5 | C6 | C7 | 120.13 | O20 | Zn23 | N1 | 100.28 | C37 | C38 | N44 | 122.35 |
| C5 | C6 | H52 | 120.05 | O20 | Zn23 | N24 | 114.67 | C39 | C38 | N44 | 117.38 |
| C7 | C6 | H52 | 119.81 | O43 | Zn23 | N1 | 110.27 | C38 | C39 | C40 | 120.4 |
| C6 | C7 | C2 | 119.84 | O43 | Zn23 | N24 | 100.32 | C38 | C39 | H63 | 119.81 |
| C6 | C7 | Cl8 | 119.13 | N1 | Zn23 | N24 | 123.7 | C40 | C39 | H63 | 119.79 |
| C2 | C7 | Cl8 | 120.95 | C25 | N24 | N45 | 116.97 | C39 | C40 | C41 | 119.15 |
| C11 | N10 | C19 | 119.34 | C25 | N24 | Zn23 | 122 | C39 | C40 | N45 | 116.34 |
| N10 | C11 | C12 | 121.14 | N45 | N24 | Zn23 | 119.67 | C41 | C40 | N45 | 124.5 |
| N10 | C11 | H53 | 118.91 | N24 | C25 | C26 | 120.52 | C40 | C41 | C42 | 119.95 |
| C12 | C11 | H53 | 119.95 | N24 | C25 | C30 | 120.32 | C40 | C41 | O43 | 121.43 |
| C11 | C12 | C13 | 119.85 | C26 | C25 | C30 | 119.15 | C42 | C41 | O43 | 118.57 |
| C11 | C12 | H54 | 120.14 | C25 | C26 | C27 | 120.16 | C41 | C42 | N33 | 118.17 |
| C13 | C12 | H54 | 120.01 | C25 | C26 | Cl32 | 120.63 | C41 | C42 | C37 | 120.03 |
| C12 | C13 | C14 | 119.31 | C27 | C26 | Cl32 | 119.01 | N33 | C42 | C37 | 121.79 |
| C12 | C13 | H55 | 120.29 | C26 | C27 | C28 | 120.04 | C41 | O43 | Zn23 | 109.59 |
| C14 | C13 | H55 | 120.4 | C26 | C27 | H57 | 119.83 | C38 | N44 | O48 | 121.36 |
| C13 | C14 | C15 | 122.04 | C28 | C27 | H57 | 120.11 | C38 | N44 | O49 | 118.94 |
| C13 | C14 | C19 | 118.8 | C27 | C28 | C29 | 120.18 | O48 | N44 | O49 | 119.69 |
| C15 | C14 | C19 | 119.15 | C27 | C28 | H58 | 119.9 | N24 | N45 | C40 | 121.3 |
| C14 | C15 | C16 | 120.37 | C29 | C28 | H58 | 119.92 |  |  |  |  |
| C14 | C15 | N21 | 122.59 | C28 | C29 | C30 | 120.03 |  |  |  |  |
| C16 | C15 | N21 | 117.05 | C28 | C29 | H59 | 120.1 |  |  |  |  |
| C15 | C16 | C17 | 120.82 | C30 | C29 | H59 | 119.87 |  |  |  |  |
| C15 | C16 | H56 | 119.56 | C29 | C30 | C25 | 120.15 |  |  |  |  |
| C17 | C16 | H56 | 119.63 | C29 | C30 | Cl31 | 118.96 |  |  |  |  |

**Table S10.** List of bond lengths for the optimized structure of ligand **CPAQ-Zn**

| Atom1 | Atom2 | Type | Length | Atom1 | Atom2 | Type | Length |
| --- | --- | --- | --- | --- | --- | --- | --- |
| N1 | C2 | Single | 1.4833 | C34 | C35 | Single | 1.5015 |
| C2 | C3 | Single | 1.5065 | C35 | C36 | Double | 1.4941 |
| C3 | C4 | Double | 1.4977 | C36 | C37 | Single | 1.5006 |
| C4 | C5 | Single | 1.4972 | C37 | C38 | Single | 1.5166 |
| C5 | C6 | Double | 1.4987 | C38 | C39 | Double | 1.4982 |
| C6 | C7 | Single | 1.4983 | C39 | C40 | Single | 1.4997 |
| C2 | C7 | Double | 1.5092 | C40 | C41 | Double | 1.5179 |
| C7 | Cl8 | Single | 1.7489 | C41 | C42 | Single | 1.5043 |
| C3 | Cl9 | Single | 1.7477 | N33 | C42 | Single | 1.47 |
| N10 | C11 | Double | 1.4625 | C37 | C42 | Double | 1.5069 |
| C11 | C12 | Single | 1.5007 | C41 | O43 | Single | 1.4545 |
| C12 | C13 | Double | 1.4937 | C38 | N44 | Single | 1.4797 |
| C13 | C14 | Single | 1.5017 | N24 | N45 | Double | 1.4392 |
| C14 | C15 | Single | 1.5153 | N45 | C40 | Single | 1.4839 |
| C15 | C16 | Double | 1.4969 | Zn23 | O43 | Single | 1.9325 |
| C16 | C17 | Single | 1.5003 | N1 | Zn23 | Single | 1.9498 |
| C17 | C18 | Double | 1.5222 | N24 | Zn23 | Single | 1.9519 |
| C18 | C19 | Single | 1.5065 | N21 | O46 | Double | 1.4285 |
| N10 | C19 | Single | 1.4701 | N21 | O47 | Double | 1.4271 |
| C14 | C19 | Double | 1.5073 | N44 | O48 | Double | 1.4285 |
| C18 | O20 | Single | 1.4571 | N44 | O49 | Double | 1.4284 |
| C15 | N21 | Single | 1.4804 | C4 | H50 | Single | 1.1293 |
| N1 | N22 | Double | 1.4375 | C5 | H51 | Single | 1.1288 |
| N22 | C17 | Single | 1.4857 | C6 | H52 | Single | 1.1288 |
| O20 | Zn23 | Single | 1.9324 | C11 | H53 | Single | 1.1289 |
| N24 | C25 | Single | 1.4847 | C12 | H54 | Single | 1.129 |
| C25 | C26 | Single | 1.5078 | C13 | H55 | Single | 1.1264 |
| C26 | C27 | Double | 1.4976 | C16 | H56 | Single | 1.1293 |
| C27 | C28 | Single | 1.4971 | C27 | H57 | Single | 1.1289 |
| C28 | C29 | Double | 1.4975 | C28 | H58 | Single | 1.1288 |
| C29 | C30 | Single | 1.4976 | C29 | H59 | Single | 1.1288 |
| C25 | C30 | Double | 1.511 | C34 | H60 | Single | 1.1289 |
| C30 | Cl31 | Single | 1.7483 | C35 | H61 | Single | 1.1289 |
| C26 | Cl32 | Single | 1.7475 | C36 | H62 | Single | 1.1267 |
| N33 | C34 | Double | 1.4617 | C39 | H63 | Single | 1.1293 |

**Table S11.** List of bond angles for the optimized structure of ligand **CPAQ-Cd**

| Atom1 | Atom2 | Atom3 | Angle | Atom1 | Atom2 | Atom3 | Angle | Atom1 | Atom2 | Atom3 | Angle |
| --- | --- | --- | --- | --- | --- | --- | --- | --- | --- | --- | --- |
| C2 | N1 | N22 | 118.82 | C16 | C17 | C18 | 118.87 | C25 | C30 | Cl31 | 120.73 |
| C2 | N1 | Cd23 | 120.76 | C16 | C17 | N22 | 116.36 | C34 | N33 | C42 | 120.78 |
| N22 | N1 | Cd23 | 118.02 | C18 | C17 | N22 | 124.72 | N33 | C34 | C35 | 120.15 |
| N1 | C2 | C3 | 121.91 | C17 | C18 | C19 | 119.57 | N33 | C34 | H60 | 119.92 |
| N1 | C2 | C7 | 118.95 | C17 | C18 | O20 | 123.02 | C35 | C34 | H60 | 119.93 |
| C3 | C2 | C7 | 119.13 | C19 | C18 | O20 | 117.3 | C34 | C35 | C36 | 119.74 |
| C2 | C3 | C4 | 119.83 | C18 | C19 | N10 | 119.18 | C34 | C35 | H61 | 120.14 |
| C2 | C3 | Cl9 | 121.41 | C18 | C19 | C14 | 120.63 | C36 | C35 | H61 | 120.12 |
| C4 | C3 | Cl9 | 118.53 | N10 | C19 | C14 | 120.16 | C35 | C36 | C37 | 119.55 |
| C3 | C4 | C5 | 120.31 | C18 | O20 | Cd23 | 111.41 | C35 | C36 | H62 | 119.98 |
| C3 | C4 | H50 | 119.84 | C15 | N21 | O46 | 119.62 | C37 | C36 | H62 | 120.47 |
| C5 | C4 | H50 | 119.82 | C15 | N21 | O47 | 120.8 | C36 | C37 | C38 | 120.83 |
| C4 | C5 | C6 | 119.88 | O46 | N21 | O47 | 119.58 | C36 | C37 | C42 | 119.54 |
| C4 | C5 | H51 | 120.05 | N1 | N22 | C17 | 125.48 | C38 | C37 | C42 | 119.63 |
| C6 | C5 | H51 | 120.06 | O20 | Cd23 | O43 | 113.06 | C37 | C38 | C39 | 119.65 |
| C5 | C6 | C7 | 120.05 | O20 | Cd23 | N1 | 95.64 | C37 | C38 | N44 | 121.06 |
| C5 | C6 | H52 | 119.96 | O20 | Cd23 | N24 | 117.05 | C39 | C38 | N44 | 119.29 |
| C7 | C6 | H52 | 119.99 | O43 | Cd23 | N1 | 112.98 | C38 | C39 | C40 | 120.71 |
| C6 | C7 | C2 | 120.3 | O43 | Cd23 | N24 | 96.69 | C38 | C39 | H63 | 119.65 |
| C6 | C7 | Cl8 | 119.18 | N1 | Cd23 | N24 | 122.45 | C40 | C39 | H63 | 119.64 |
| C2 | C7 | Cl8 | 120.47 | C25 | N24 | N45 | 118.75 | C39 | C40 | C41 | 118.68 |
| C11 | N10 | C19 | 120.89 | C25 | N24 | Cd23 | 121.66 | C39 | C40 | N45 | 117.04 |
| N10 | C11 | C12 | 120.09 | N45 | N24 | Cd23 | 117.74 | C41 | C40 | N45 | 124.28 |
| N10 | C11 | H53 | 119.95 | N24 | C25 | C26 | 122.21 | C40 | C41 | C42 | 119.32 |
| C12 | C11 | H53 | 119.96 | N24 | C25 | C30 | 119 | C40 | C41 | O43 | 122.43 |
| C11 | C12 | C13 | 119.74 | C26 | C25 | C30 | 118.8 | C42 | C41 | O43 | 118.15 |
| C11 | C12 | H54 | 120.13 | C25 | C26 | C27 | 119.85 | C41 | C42 | N33 | 119.5 |
| C13 | C12 | H54 | 120.13 | C25 | C26 | Cl32 | 121.73 | C41 | C42 | C37 | 120.27 |
| C12 | C13 | C14 | 119.63 | C27 | C26 | Cl32 | 118.13 | N33 | C42 | C37 | 120.21 |
| C12 | C13 | H55 | 119.89 | C26 | C27 | C28 | 120.4 | C41 | O43 | Cd23 | 108.45 |
| C14 | C13 | H55 | 120.48 | C26 | C27 | H57 | 119.78 | C38 | N44 | O48 | 120.63 |
| C13 | C14 | C15 | 120.77 | C28 | C27 | H57 | 119.78 | C38 | N44 | O49 | 119.65 |
| C13 | C14 | C19 | 119.49 | C27 | C28 | C29 | 119.8 | O48 | N44 | O49 | 119.7 |
| C15 | C14 | C19 | 119.73 | C27 | C28 | H58 | 120.09 | N24 | N45 | C40 | 125.01 |
| C14 | C15 | C16 | 119.77 | C29 | C28 | H58 | 120.08 |  |  |  |  |
| C14 | C15 | N21 | 121.22 | C28 | C29 | C30 | 120.13 |  |  |  |  |
| C16 | C15 | N21 | 119 | C28 | C29 | H59 | 119.92 |  |  |  |  |
| C15 | C16 | C17 | 121.07 | C30 | C29 | H59 | 119.94 |  |  |  |  |
| C15 | C16 | H56 | 119.46 | C29 | C30 | C25 | 120.35 |  |  |  |  |
| C17 | C16 | H56 | 119.46 | C29 | C30 | Cl31 | 118.88 |  |  |  |  |

**Table S12.** List of bond lengths for the optimized structure of ligand **CPAQ-Cd**

| Atom1 | Atom2 | Type | Length | Atom1 | Atom2 | Type | Length |
| --- | --- | --- | --- | --- | --- | --- | --- |
| N1 | C2 | Single | 1.5209 | C34 | C35 | Single | 1.5389 |
| C2 | C3 | Single | 1.5502 | C35 | C36 | Double | 1.5374 |
| C3 | C4 | Double | 1.541 | C36 | C37 | Single | 1.54 |
| C4 | C5 | Single | 1.5385 | C37 | C38 | Single | 1.5411 |
| C5 | C6 | Double | 1.5375 | C38 | C39 | Double | 1.5388 |
| C6 | C7 | Single | 1.5402 | C39 | C40 | Single | 1.5415 |
| C2 | C7 | Double | 1.5475 | C40 | C41 | Double | 1.5575 |
| C7 | Cl8 | Single | 1.7609 | C41 | C42 | Single | 1.5405 |
| C3 | Cl9 | Single | 1.7621 | N33 | C42 | Single | 1.5115 |
| N10 | C11 | Double | 1.5107 | C37 | C42 | Double | 1.5401 |
| C11 | C12 | Single | 1.5382 | C41 | O43 | Single | 1.5129 |
| C12 | C13 | Double | 1.5372 | C38 | N44 | Single | 1.5121 |
| C13 | C14 | Single | 1.5401 | N24 | N45 | Double | 1.4857 |
| C14 | C15 | Single | 1.5405 | N45 | C40 | Single | 1.5257 |
| C15 | C16 | Double | 1.5376 | Cd23 | O43 | Single | 2.1348 |
| C16 | C17 | Single | 1.5413 | N1 | Cd23 | Single | 2.1414 |
| C17 | C18 | Double | 1.5613 | N24 | Cd23 | Single | 2.1458 |
| C18 | C19 | Single | 1.541 | N21 | O46 | Double | 1.4805 |
| N10 | C19 | Single | 1.5119 | N21 | O47 | Double | 1.4784 |
| C14 | C19 | Double | 1.5402 | N44 | O48 | Double | 1.4794 |
| C18 | O20 | Single | 1.5139 | N44 | O49 | Double | 1.4802 |
| C15 | N21 | Single | 1.5125 | C4 | H50 | Single | 1.1399 |
| N1 | N22 | Double | 1.4837 | C5 | H51 | Single | 1.1401 |
| N22 | C17 | Single | 1.5265 | C6 | H52 | Single | 1.1402 |
| O20 | Cd23 | Single | 2.1364 | C11 | H53 | Single | 1.14 |
| N24 | C25 | Single | 1.5248 | C12 | H54 | Single | 1.14 |
| C25 | C26 | Single | 1.5528 | C13 | H55 | Single | 1.1379 |
| C26 | C27 | Double | 1.5408 | C16 | H56 | Single | 1.1399 |
| C27 | C28 | Single | 1.5373 | C27 | H57 | Single | 1.1398 |
| C28 | C29 | Double | 1.5365 | C28 | H58 | Single | 1.1403 |
| C29 | C30 | Single | 1.5398 | C29 | H59 | Single | 1.1399 |
| C25 | C30 | Double | 1.5497 | C34 | H60 | Single | 1.14 |
| C30 | Cl31 | Single | 1.7609 | C35 | H61 | Single | 1.14 |
| C26 | Cl32 | Single | 1.7621 | C36 | H62 | Single | 1.1386 |
| N33 | C34 | Double | 1.511 | C39 | H63 | Single | 1.14 |


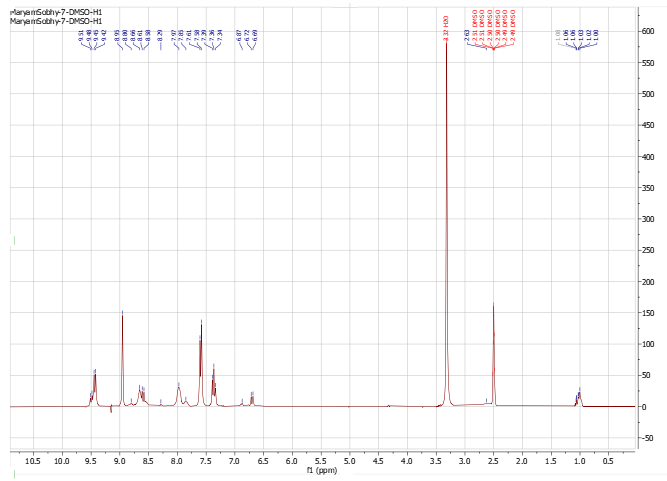


**Fig. S1.** 1H-NMR spectrum of **CPAQ-Zn** complex in d6-DMSO


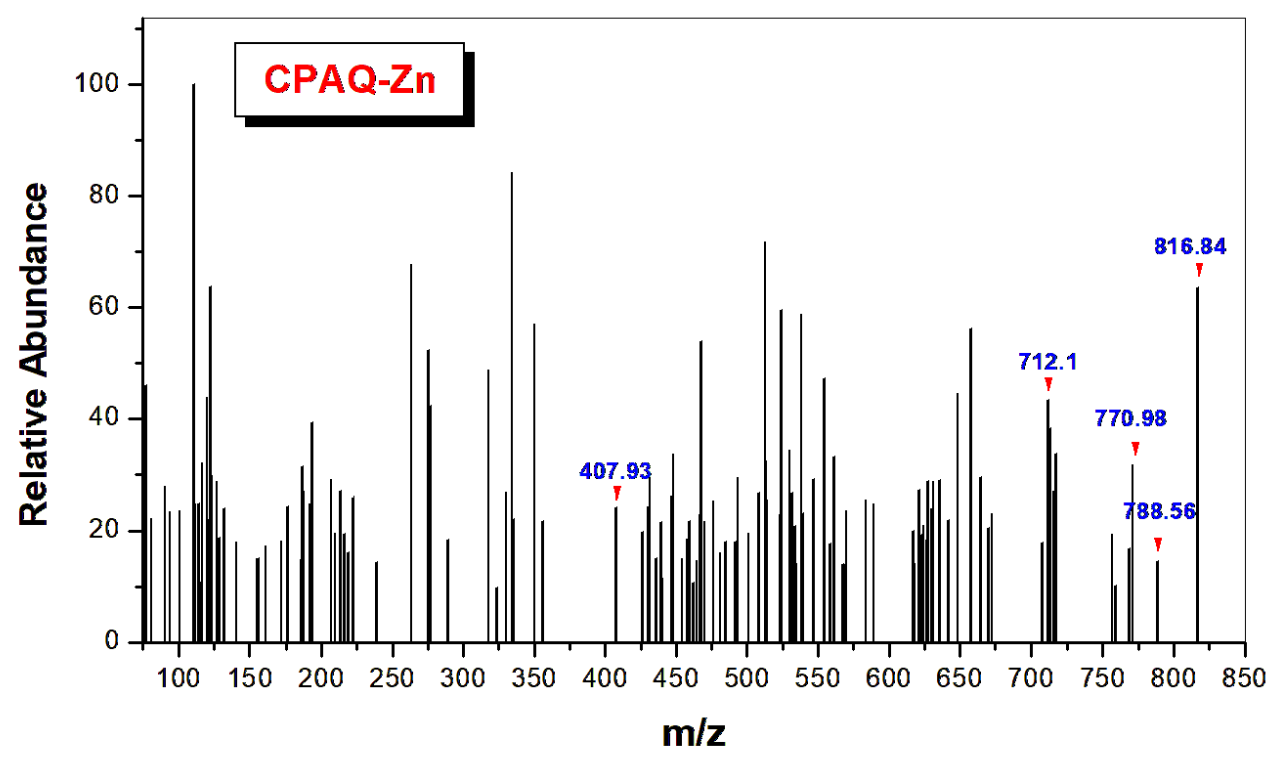


**Fig. S2.** Mass spectrum of **CPAQ-Zn** complex


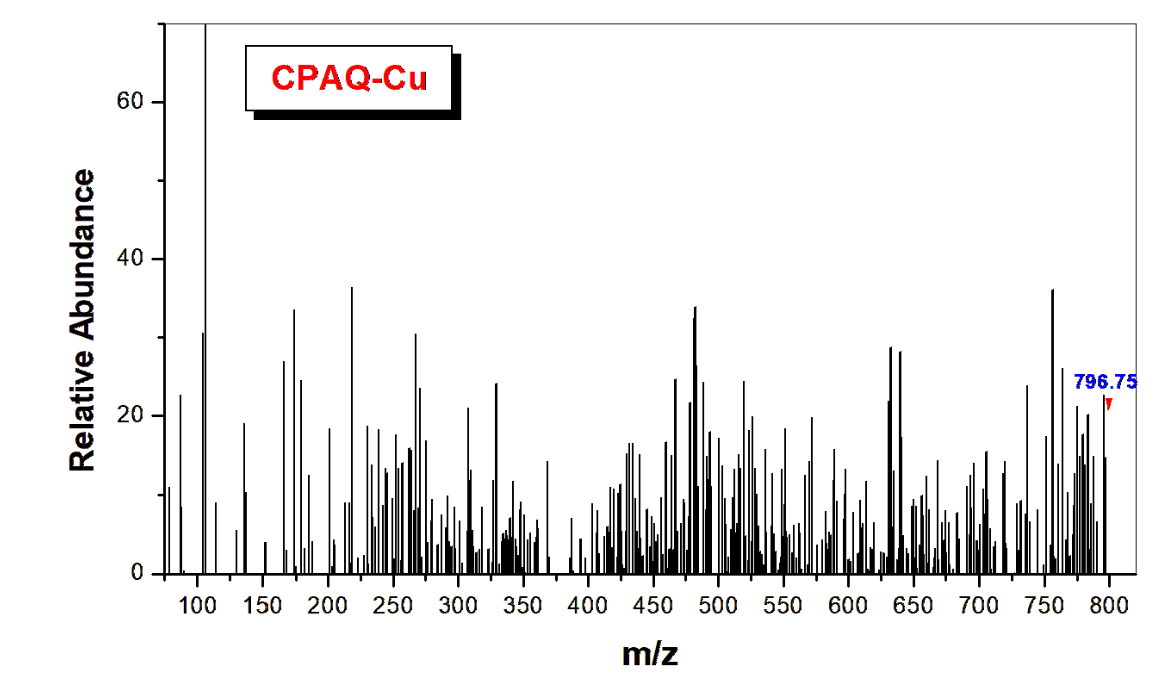


**Fig. S3.** Mass spectrum of **CPAQ-Zn** complex


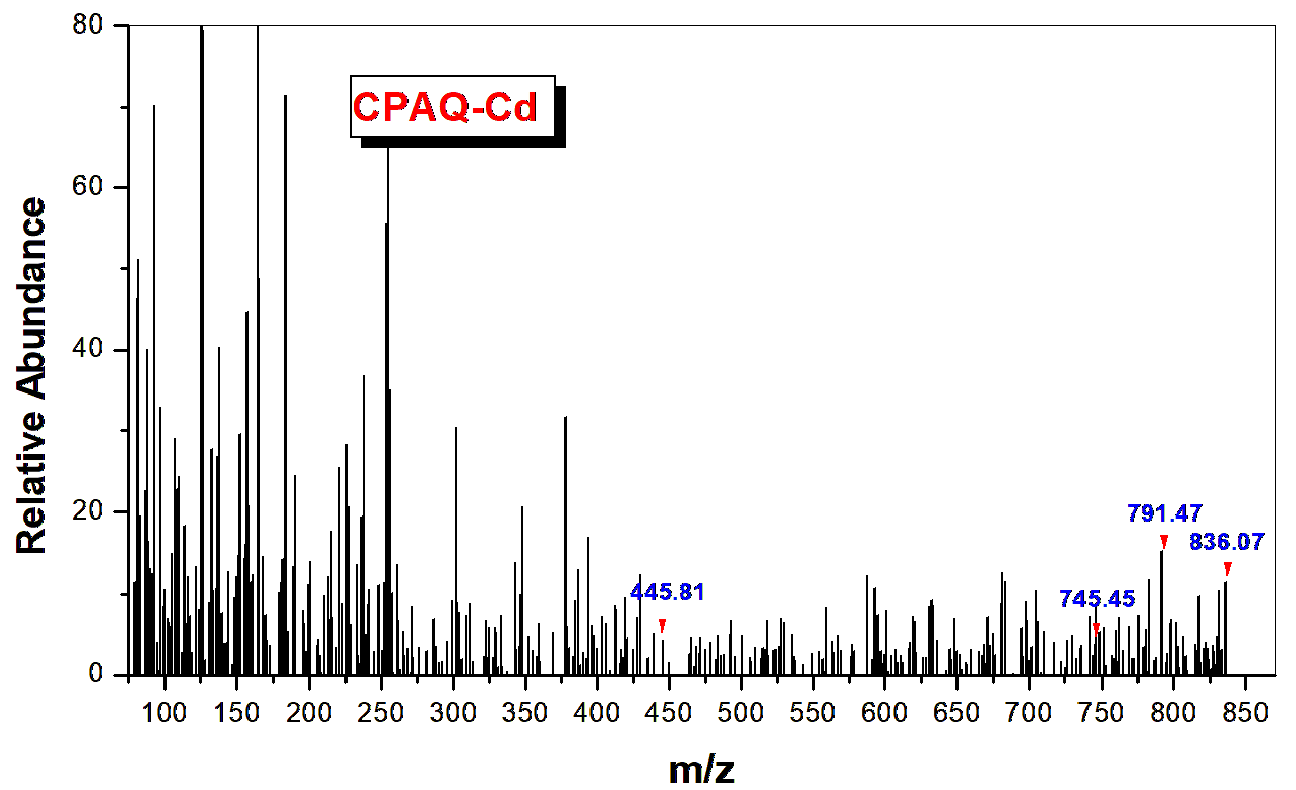


**Fig. S4.** Mass spectrum of **CPAQ-Cd** complex


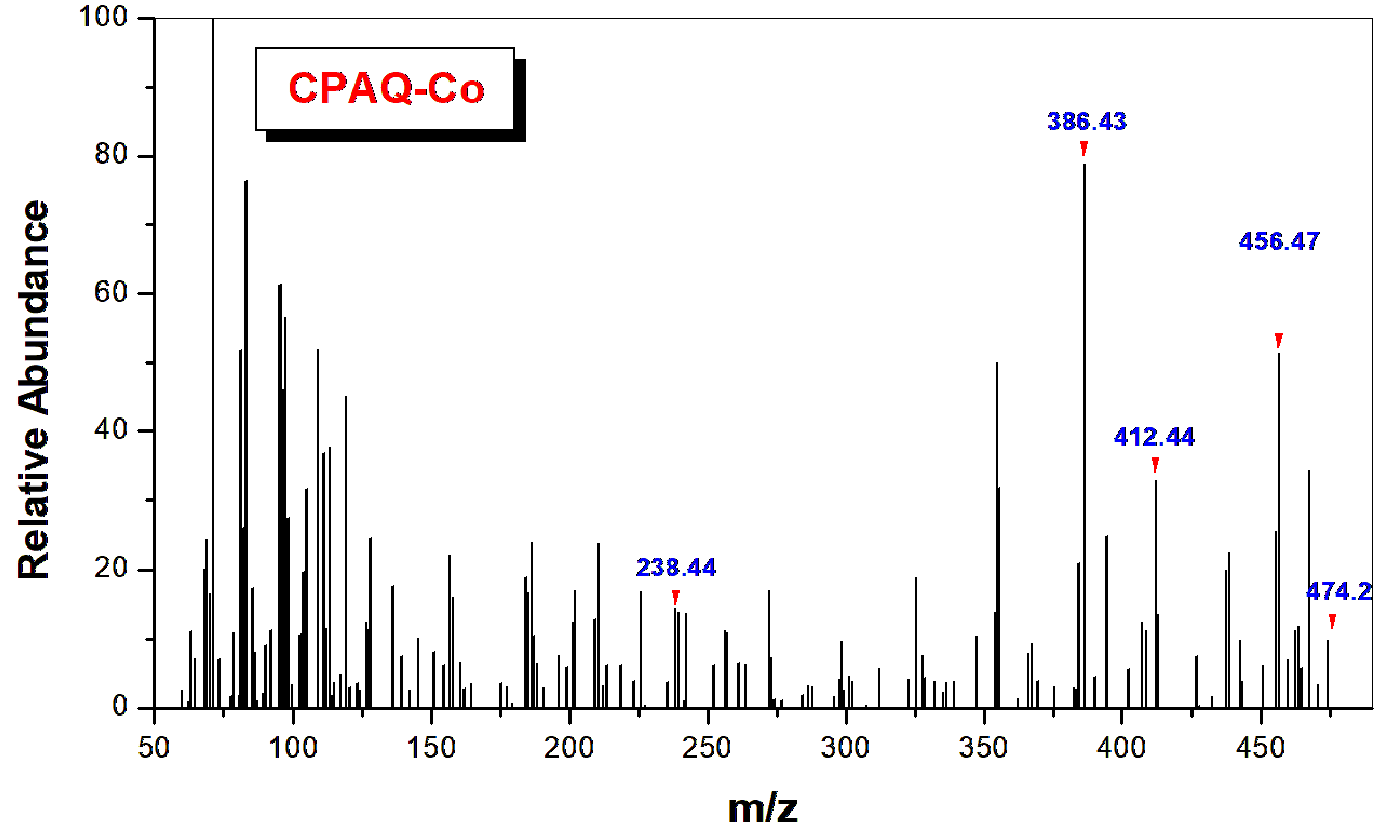


**Fig. S5.** Mass spectrum of **CPAQ-Co** complex





**Scheme S1.** Fragmentation pathways of the organic ligand **CPAQ**





**Scheme S2.** Fragmentation pathways of the organic ligand **CPAQ-Zn**





**Scheme S3.** Fragmentation pathways of the organic ligand **CPAQ-Cu**





**Scheme S4.** Fragmentation pathways of the organic ligand **CPAQ-Cd**



**Scheme S5.** Fragmentation pathways of the organic ligand **CPAQ-Ni**





**Scheme S6.** Fragmentation pathways of the organic ligand **CPAQ-Co**
